# Supplementary material for: Altering the spectroscopy, electronic structure, and bonding of organometallic curium(III) upon coordination of 4,4′−bipyridine
Source: Nat Commun. 2023 Jun 24;14:3774. doi: 10.1038/s41467-023-39481-7 (PMC10290646; doi:10.1038/s41467-023-39481-7)
Supplement: Supplementary file 1 — Supplementary Information [file 41467_2023_39481_MOESM1_ESM.pdf]

Supplementary Information

Altering the Spectroscopy, Electronic Structure, and Bonding of Organometallic Curium(III) Upon Coordination of 4,4′−bipyridine

Brian N. Long,[1] María J. Beltrán-Leíva,[1] Joseph M. Sperling,[1] Todd N. Poe,[1] Cristian Celis-Barros,*[,1,2] Thomas E. Albrecht-Schönzart*[,1,2]

[1]Department of Chemistry and Biochemistry, Florida State University, 95 Chieftan Way, Tallahassee, Florida 32306

[2]Department of Chemistry and Nuclear Science & Engineering Center, Colorado School of Mines, Golden, Colorado 80401

*To whom correspondence should be addressed: talbrechtschoenzart@gmail.com, Ccelisbarros@mines.edu

Key Words: actinides, curium, cyclopentadienyl ligands, organoactinide, organometallic

Supplementary Information

Table of Contents

Supplementary Note 1: Characterization Methods	3
Supplementary Note 2: Synthesis Pictures	5
Supplementary Note 3: Structural Details	10
Supplementary Note 4: Solid-State Absorption and Photoluminescence	17
Supplementary Note 5: Solution Phase Absorption Spectra	26
Supplementary Note 6: Computational Details	33
Supplementary Note 7: Additional Theoretical Results	35
Supplementary Note 8: References	61

Supplementary Note 1: Characterization Methods

Crystallographic Details

Crystals were collected in on a glass slide in Parabar 10312 oil, closed in a petri dish, and taped shut to prevent air exposure while transporting to a microscope. Single crystals were mounted on 75 μm MiTeGen loops and placed under a stream of N_2(g). Diffraction data was collected on a Bruker D8 Quest X-ray Diffractometer using a IμS X-ray source (Mo K_α; λ = 0.71073 Å). Unit cell determination and data collection of 1−Sm was completed at 120 K, while 1−Cm and 1−Gd were completed at 100 K. Centering, unit cell collection, and frame integrations were completed using APEXIII. Characterization and refinements were performed through SHELXTL utilizing the OLEX2 GUI. [1,2] Structural and crystallographic details can be found in Tables S1 - S6. Cif files for each crystal can be found under CCDC Deposition numbers: 1−Sm (2236796), 1−Gd (2236795), and 1−Cm (2236794).

Solid State Absorption Spectroscopy and Photoluminescence

In an argon atmosphere glovebox, single crystals were collected under Parabar 10312 oil on a glass slide, closed into a petri dish, and taped to prevent air exposure while transporting to the instrument. Photoluminescence and absorption spectra were performed using a CRAIC Technologies 20/20 PV™ Microspectrophotometer with a 100 W mercury light source and 75 W xenon lamp for photoluminescence and transmission, respectively. Data collection of 1−Cm was collected at room temperature (23 °C) and −180 °C. A Linkam LTS420 stage was used purged with N_2 for 5 minutes and cooled to −180 °C at a rate of 5 °C/min. Absorption spectra were

collected from 320 nm to 1,700 nm and photoluminescence was collected from 320 nm to 800 nm using excitation sources of 365 nm and 420 nm.

Solution Phase Absorption Spectroscopy

In an argon glovebox, samples were filtered in toluene and transferred to a Starna GL14-C screw cap cell. Absorption spectra were performed at room temperature on a Cary 6000i series UV/Vis/NIR spectrophotometer from 250 nm to 1700 nm with a pathlength of 0.4 cm. Spectra of 1−Sm and 1−Gd were measured again after being exposed to air for 24 hours while spectra of 1−Cm were collected each hour over a 48 hour period.

Supplementary Note 2: Synthesis Pictures

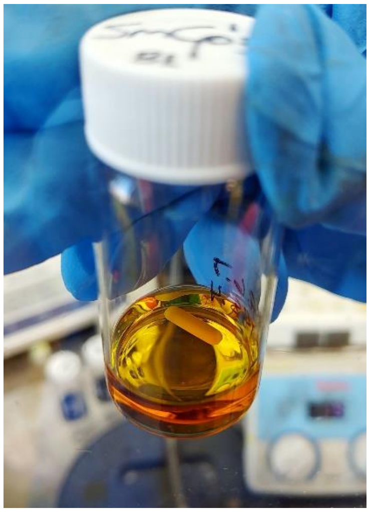

a

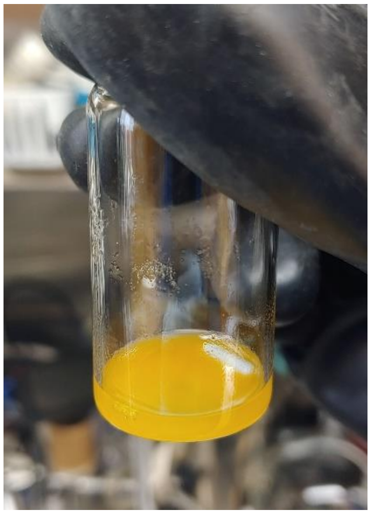

b

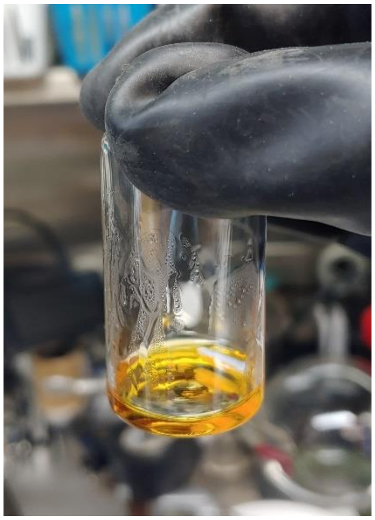

c

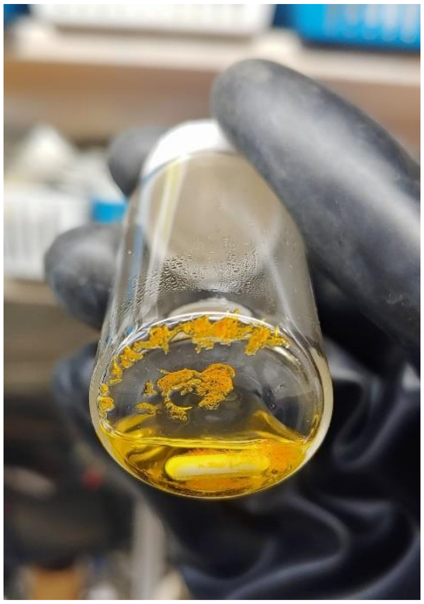

d

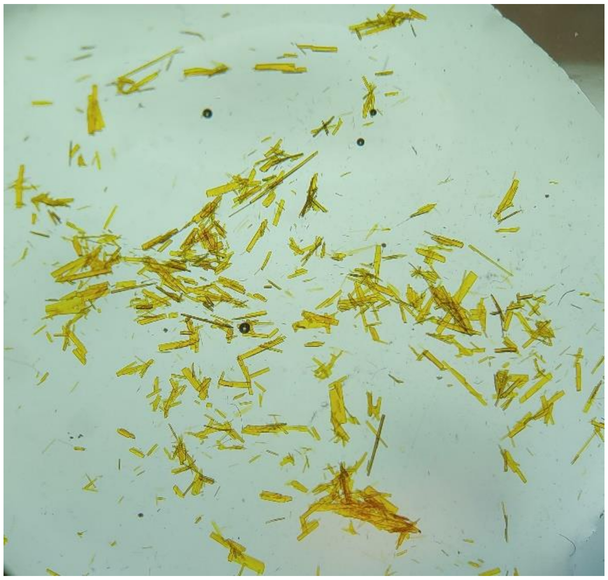

e

Supplementary Figure 1. **Reaction photos of 1−Sm**. **a**. A sample of Cp'_3Sm in hexane. **b**. $(Cp'_3Sm)_2(\mu-4,4'-bpy)$ precipitate in toluene. **c**. Solution of $(Cp'_3Sm)_2(\mu-4,4'-bpy)$ at 120 °C. **d**. Crystals of $(Cp'_3Sm)_2(\mu-4,4'-bpy)$ grown by cooling to room temperature. **e**. Crystals used for single-crystal X-ray diffraction.

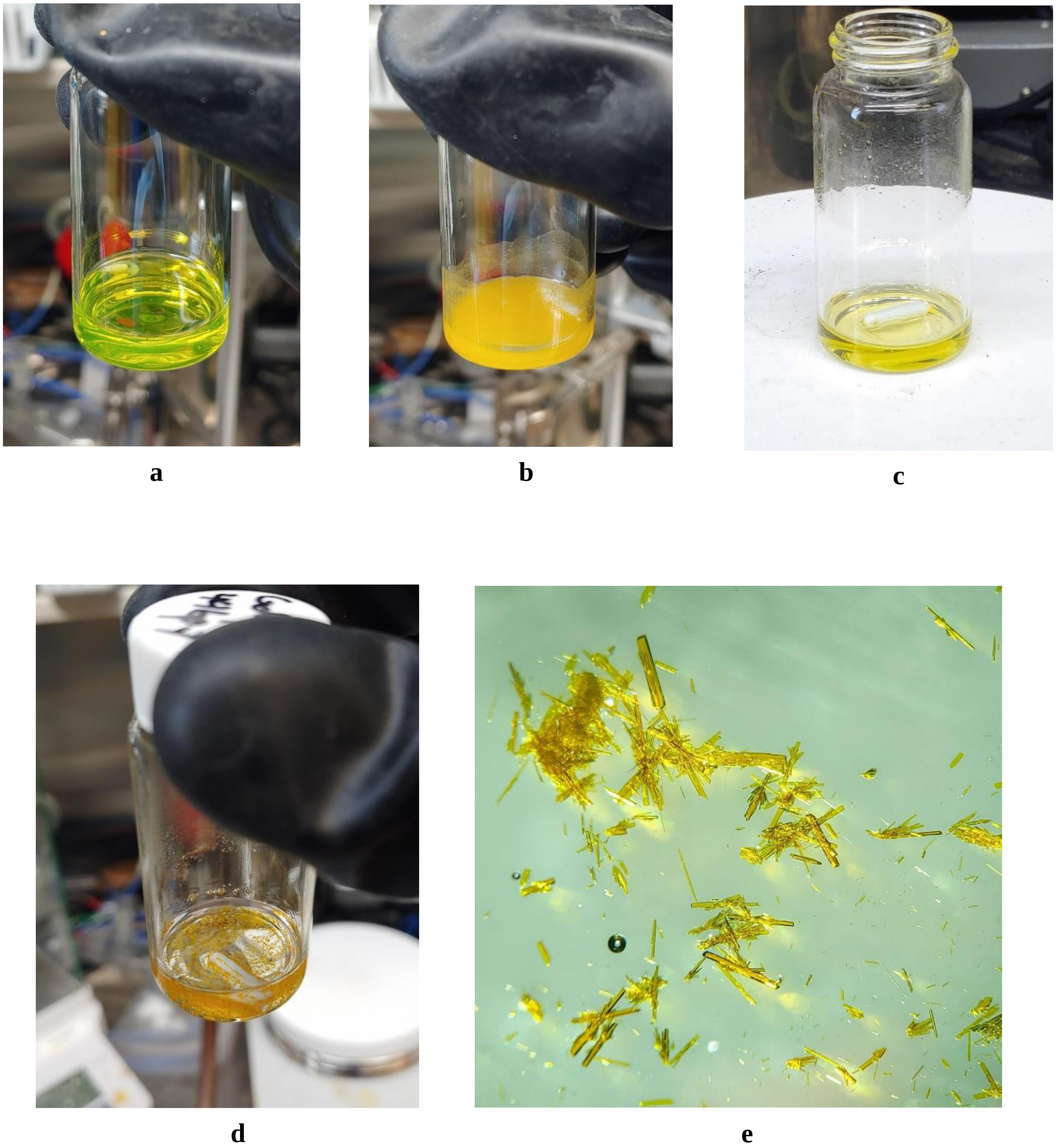

Supplementary Figure 2. **Reaction photos of 1−Gd**. **a**. A solution of Cp′$_3$Gd in hexane. **b**. (Cp′$_3$Gd)$_2$(μ−4,4′−bpy) precipitate in toluene. **c**. Solution of (Cp′$_3$Gd)$_2$(μ−4,4′−bpy) heated to 120 °C. **d**. Crystals of (Cp′$_3$Sm)$_2$(μ−4,4′−bpy) grown by cooling to room temperature. **e**. Crystals used for single-crystal X-ray diffraction.

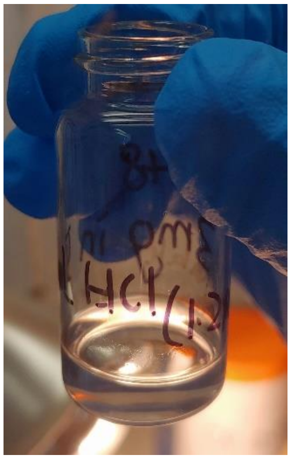

a

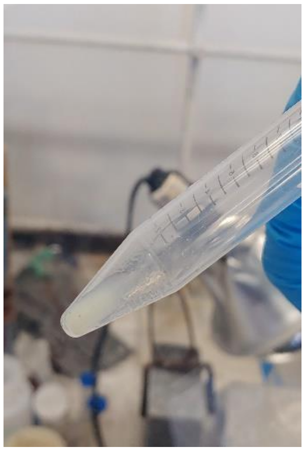

b

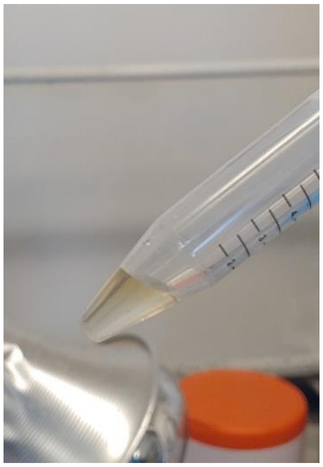

c

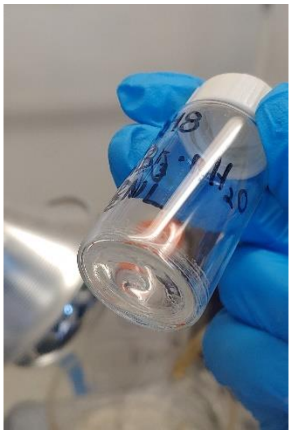

d

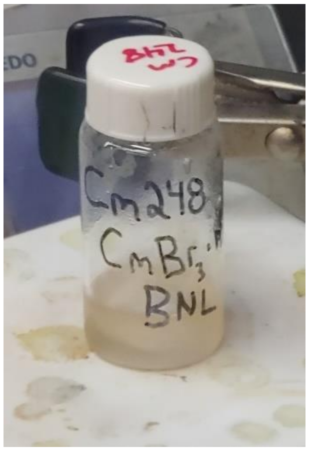

e

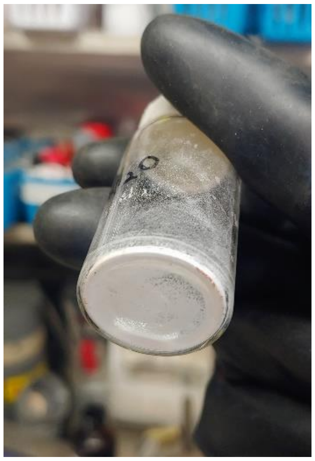

f

Supplementary Figure 3. **Reaction photos of 1−Cm**. **a**. A stock solution of Cm^{3+} (3.3 mg) in HCl (2 M) **b**. The pellet of $Cm(OH)_3$ after rinsing with H_2O. **c**. Cm dissolved in HBr (8.77 M) **d**. Dried residue of $CmBr_3(H_2O)_n$ after washing with OEt_2. **e**. Slurry of $CmBr_3(DME)_n$ in DME and TMS−Br. **f**. Dried powder of $CmBr_3(DME)_n$.

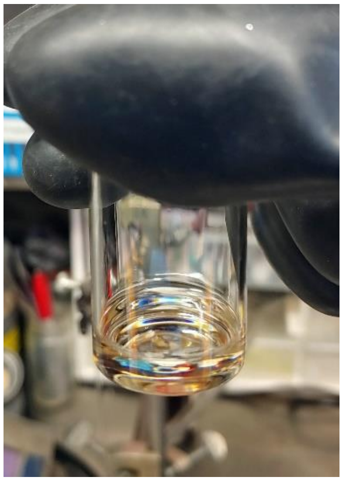

g

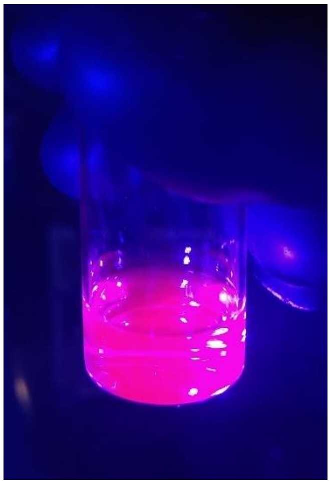

h

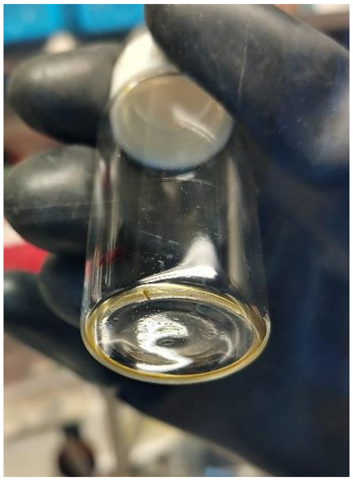

i

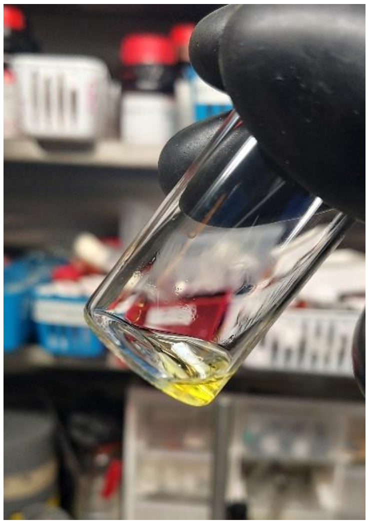

j

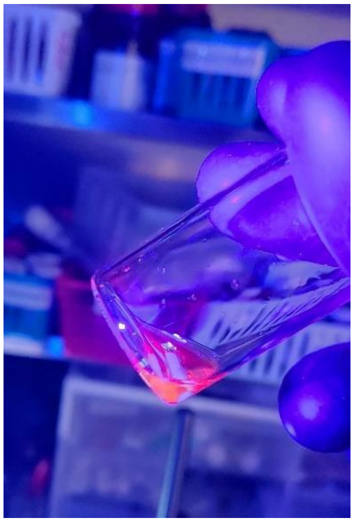

k

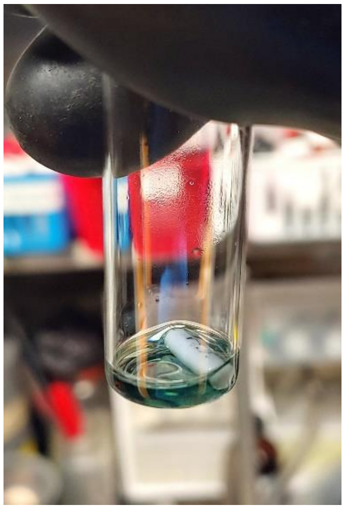

l

Supplementary Figure 4. **Reaction photos of 1−Cm**. **g**. A putative solution Cp′$_3$Cm in hexane. **h**. The putative solution of Cp′$_3$Cm glowing under a blacklight. **i**. Dried Cp′$_3$Cm forming an oil and small crystal clusters. **j**. Solution of (Cp′$_3$Cm)$_2$(μ−4,4′−bpy) in toluene. **k**. Solution of (Cp′$_3$Cm)$_2$(μ−4,4′−bpy) glowing under a blacklight. **l**. After heating to 120 °C, the yellow solution of (Cp′$_3$Cm)$_2$(μ−4,4′−bpy) turned green upon slowly cooling to room temperature.

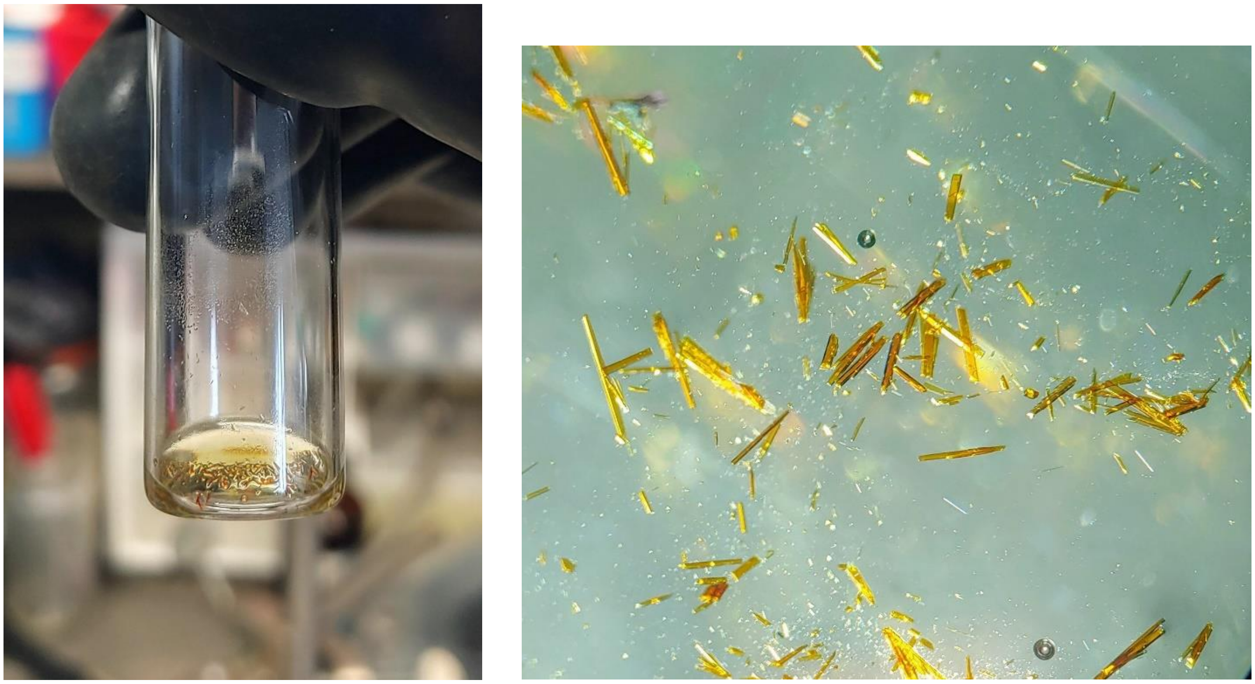

m **n**

Supplementary Figure 5. **Reaction photos of 1−Cm**. **m**. The solution turned yellow again overnight and crystals of $(Cp'_3Cm)_2(\mu{-}4,4'{-}bpy)$ suitable for single-crystal X-ray diffraction were grown at room temperature. **n**. A batch of crystals used for single-crystal X-ray diffraction.

Supplementary Note 3: Structural Details

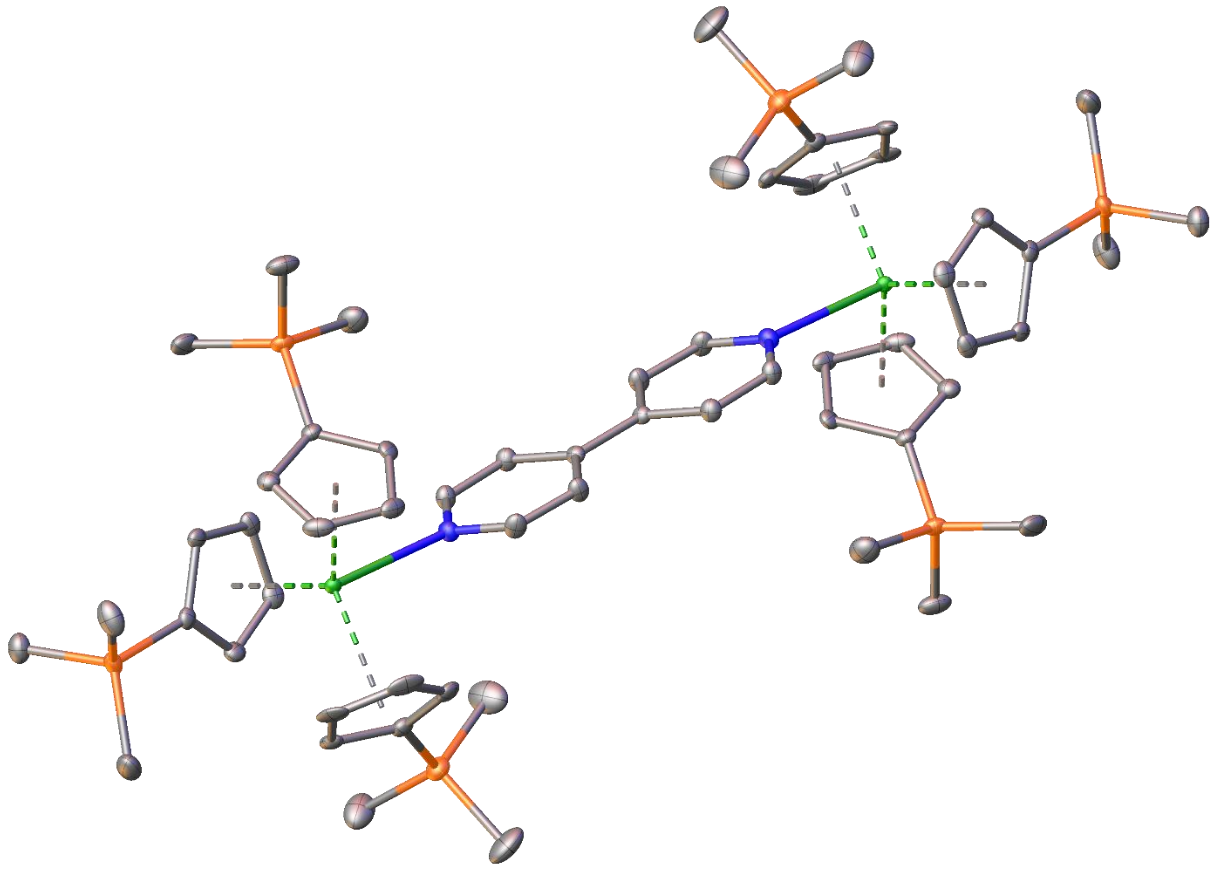

Supplementary Figure 6. **Structure of 1−Sm**. Crystal structure of $(Cp'_3Sm)_2(\mu{-}4,4'{-}bpy)$, 1−Sm. Green = Samarium, Blue = Nitrogen, Orange = Silicon, Gray = Carbon. Hydrogen has been omitted for clarity. Thermal ellipsoids are models at 50% probability.

Supplementary Table 1: Crystal data and structure refinement for 1−Sm.

Deposition Number	2236796
Empirical formula	$C_{58}H_{86}Sm_2N_2Si_6$
Formula weight (g/mol)	1280.52
Temperature/K	120
Crystal system	triclinic
Space group	$P\bar{1}$
a/Å	9.4874(13)
b/Å	11.3744(16)
c/Å	14.808(2)
α/°	99.424(3)
β/°	101.419(3)
γ/°	95.638(3)
Volume/Å^3	1531.1(4)
Z	1
ρ_{calc}g/cm^3	1.389
μ/mm^{-1}	2.052
F(000)	656.0
Crystal size/mm^3	0.675 × 0.453 × 0.158
Radiation	MoK_α (λ = 0.71073 Å)
2Θ range for data collection/°	4.712 to 55.23
Index ranges	−12 ≤ h ≤ 12, −14 ≤ k ≤ 14, −14 ≤ l ≤ 19
Reflections collected	13642
Independent reflections	7025 [R_{int} = 0.0347, R_{sigma} = 0.0550]
Data/restraints/parameters	7025/0/316
Goodness-of-fit on F^2	1.042
Final R indexes [I ≥ 2σ (I)]	R_1 = 0.0333, wR_2 = 0.0757
Final R indexes [all data]	R_1 = 0.0412, wR_2 = 0.0789
Largest diff. peak/hole / e Å^{-3}	1.19/−1.91

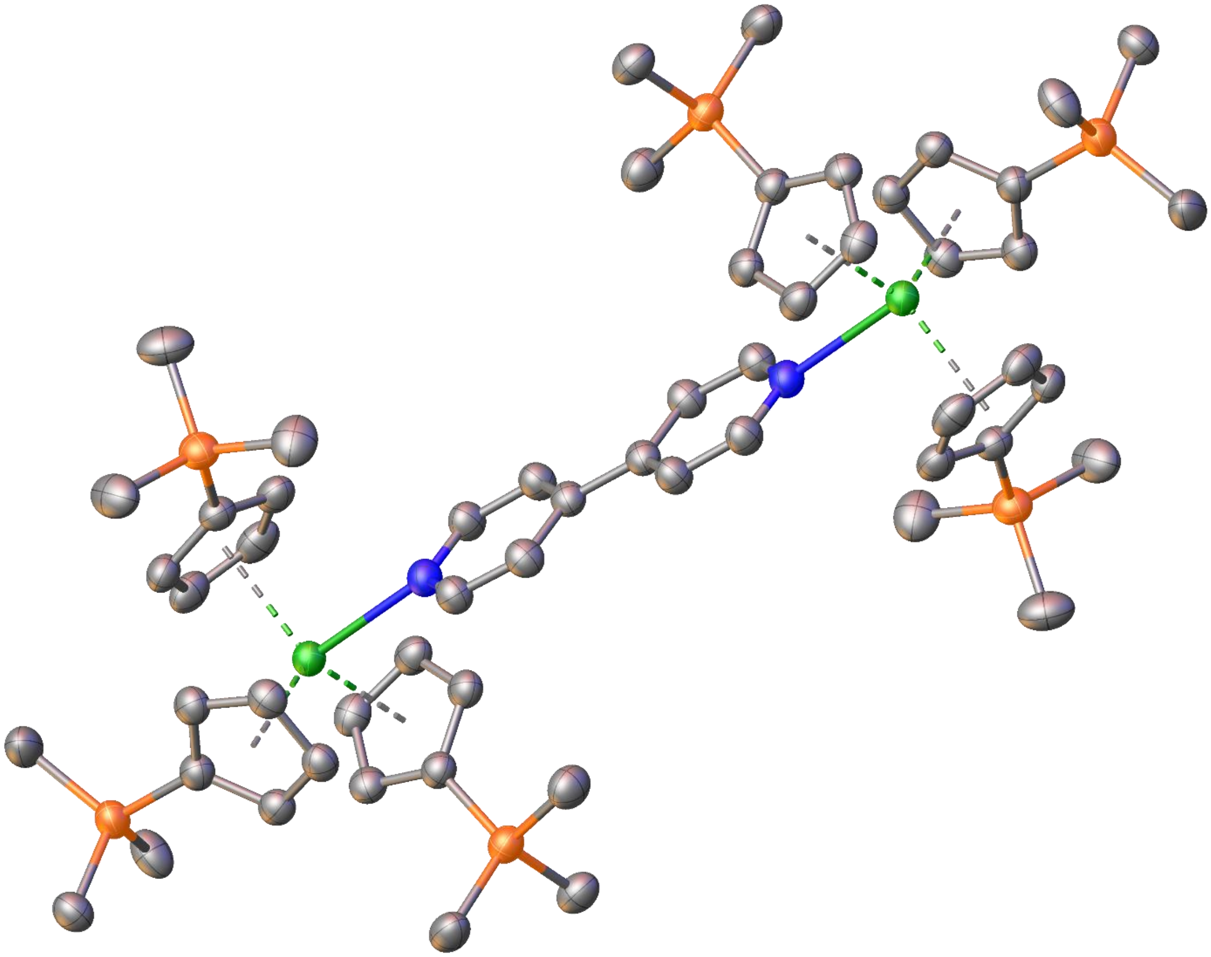

Supplementary Figure 7. **Structure of 1−Gd**. Crystal structure of $(Cp'_3Gd)_2(\mu{-}4,4'{-}bpy)$, 1−Gd. Green = Gadolinium, Blue = Nitrogen, Orange = Silicon, Gray = Carbon. Hydrogen has been omitted for clarity. Thermal ellipsoids are models at 50% probability.

**Supplementary Table 2**: Crystal data and structure refinement for 1−Gd.	
Deposition Number	2236795
Empirical formula	$C_{58}H_{86}Gd_2N_2Si_6$
Formula weight (g/mol)	1294.32
Temperature/K	100
Crystal system	triclinic
Space group	$P\bar{1}$
a/Å	9.4868(8)
b/Å	11.3583(11)
c/Å	14.7385(15)
α/°	99.336(4)
β/°	101.605(3)
γ/°	95.620(3)
Volume/Å^3	1521.0(3)
Z	1
ρ_{calc}g/cm^3	1.413
μ/mm^{-1}	2.316
F(000)	660.0
Crystal size/mm^3	0.487 × 0.169 × 0.115
Radiation	MoK_α (λ = 0.71073 Å)
2Θ range for data collection/°	4.712 to 54.988
Index ranges	$-10 \le h \le 12$, $-14 \le k \le 14$, $-19 \le l \le 18$
Reflections collected	33140
Independent reflections	6966 [R_{int} = 0.0684, R_{sigma} = 0.0482]
Data/restraints/parameters	6966/0/316
Goodness-of-fit on F^2	1.056
Final R indexes [I ≥ 2σ (I)]	R_1 = 0.0385, wR_2 = 0.0945
Final R indexes [all data]	R_1 = 0.0437, wR_2 = 0.0982
Largest diff. peak/hole / e Å^{-3}	1.52/−0.52

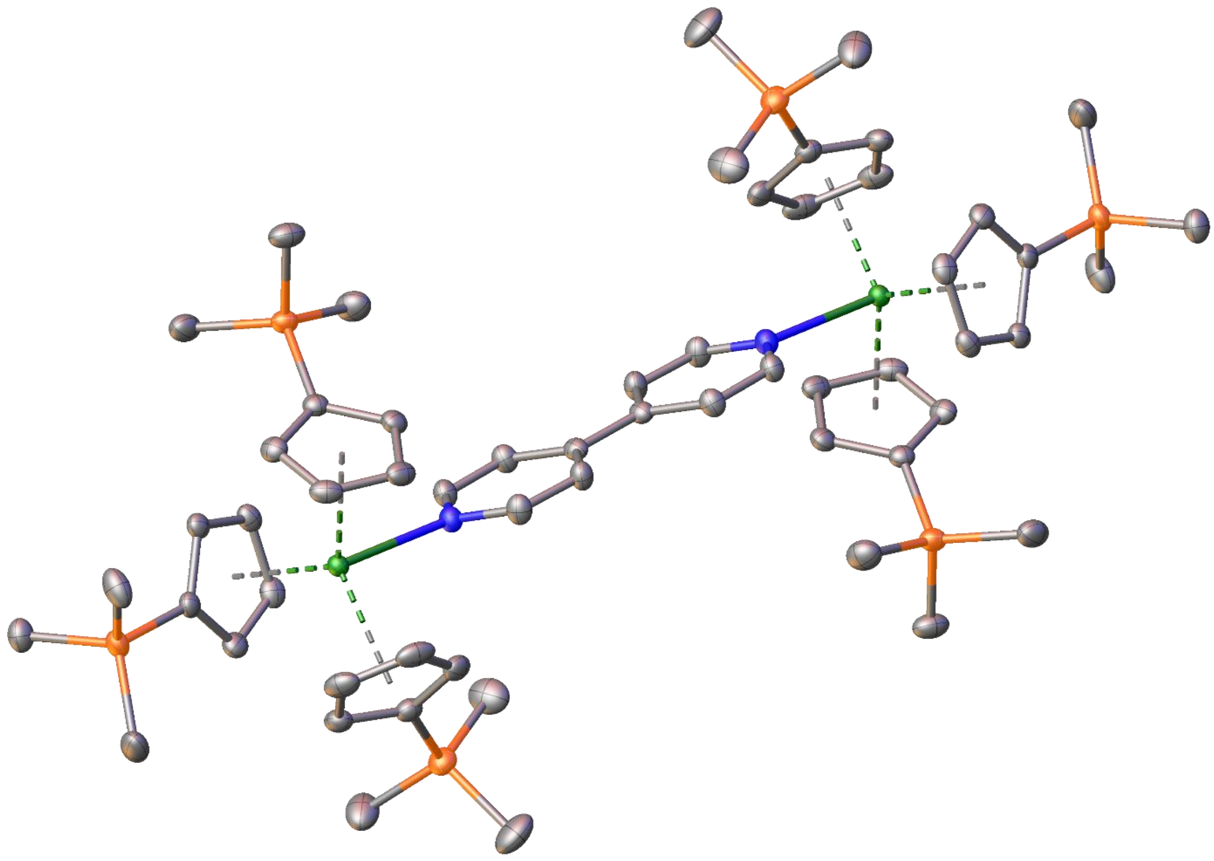

Supplementary Figure 8. **Structure of 1−Cm**. Crystal structure of $(Cp'_3Cm)_2(\mu{-}4,4'{-}bpy)$, 1−Cm. Green = Curium, Blue = Nitrogen, Orange = Silicon, Gray = Carbon. Hydrogen has been omitted for clarity. Thermal ellipsoids are models at 50% probability.

Supplementary Table 3: Crystal data and structure refinement for 1−Cm.

Deposition Number	2236794
Empirical formula	$C_{58}H_{86}Cm_2N_2Si_6$
Formula weight (g/mol)	1475.85
Temperature/K	100
Crystal system	triclinic
Space group	$P\bar{1}$
a/Å	9.4771(3)
b/Å	11.3592(4)
c/Å	14.7868(6)
α/°	99.2120(10)
β/°	101.6140(10)
γ/°	95.5520(10)
Volume/Å^3	1525.38(10)
Z	1
ρ_{calc}g/cm^3	1.598
μ/mm^{-1}	2.807
F(000)	724.0
Crystal size/mm^3	0.387 × 0.156 × 0.087
Radiation	MoK$_\alpha$ (λ = 0.71073 Å)
2Θ range for data collection/°	5.358 to 55.008
Index ranges	−12 ≤ h ≤ 11, −14 ≤ k ≤ 14, −19 ≤ l ≤ 19
Reflections collected	47077
Independent reflections	6992 [R_{int} = 0.0448, R_{sigma} = 0.0260]
Data/restraints/parameters	6992/0/348
Goodness-of-fit on F^2	1.064
Final R indexes [I ≥ 2σ (I)]	R_1 = 0.0146, wR_2 = 0.0336
Final R indexes [all data]	R_1 = 0.0160, wR_2 = 0.0342
Largest diff. peak/hole / e Å^{-3}	0.57/−0.51

Supplementary Table 4. Structural data for 1−Sm, 1−Gd, and 1−Cm. Additional information for previously reported structures of Cp′$_3$Ce(py) (Ce), [3] (Cp′$_3$Nd)$_2$(μ−4,4′−bpy) (Nd), [4] (Cp″$_3$Th)$_2$(μ−4,4′−bpy) (Th), [5] (Cp′$_3$U)$_2$(μ−4,4′−bpy) (U), [6] and (Cp′$_3$Am)$_2$(μ−4,4′−bpy) (Am) [4] is provided for further comparison.

**Bond**	**Length (Å)**							
**Compound**	**Ce**	**Nd**	**1−Sm**	**1−Gd**	**Th**	**U**	**Am**	**1−Cm**
**M1−N1**	**2.704(4)**	**2.6482(16)**	**2.626(3)**	**2.592(3)**	**2.362(4)**	**2.626(7)**	**2.618(2)**	**2.5962(16)**
**M1−Cent1**	2.573(5)	2.535(2)	2.496(3)	2.495(4)	2.606(5)	2.551(10)	2.544(3)	2.538(2)
**M1−Cent2**	2.574(5)	2.561(2)	2.537(3)	2.477(4)	2.578(5)	2.521(10)	2.521(3)	2.498(2)
**M1−Cent3**	2.587(5)	2.542(2)	2.515(3)	2.523(4)	2.597(5)	2.549(10)	2.506(3)	2.516(2)
**M1−C1**	2.843(5)	2.8000(19)	2.780(3)	2.790(3)	2.881(5)	2.902(9)	2.899(3)	2.9029(19)
**M1−C2**	2.830(5)	2.8338(19)	2.808(3)	2.742(3)	2.912(5)	2.799(9)	2.818(3)	2.835(2)
**M1−C3**	2.823(5)	2.8093(19)	2.775(3)	2.715(4)	2.923(5)	2.759(9)	2.739(3)	2.757(2)
**M1−C4**	2.849(5)	2.7751(18)	2.740(3)	2.788(4)	2.831(5)	2.774(9)	2.768(3)	2.728(2)
**M1−C5**	2.843(5)	2.7722(18)	2.748(3)	2.808(4)	2.800(5)	2.865(9)	2.844(3)	2.815(2)
**M1−C9**	2.847(5)	2.8660(19)	2.890(3)	2.754(4)	2.815(4)	2.798(9)	2.813(3)	2.7718(19)
**M1−C10**	2.863(5)	2.8296(19)	2.806(3)	2.796(4)	2.815(4)	2.763(9)	2.831(3)	2.743(2)
**M1−C11**	2.851(5)	2.7621(19)	2.734(3)	2.770(4)	2.877(4)	2.780(9)	2.811(3)	2.746(2)
**M1−C12**	2.816(5)	2.7877(19)	2.761(3)	2.724(4)	2.872(4)	2.791(8)	2.744(3)	2.786(2)
**M1−C13**	2.825(5)	2.860(2)	2.841(3)	2.719(4)	2.843(4)	2.840(9)	2.763(3)	2.816(2)
**M1−C17**	2.924(5)	2.835(2)	2.811(3)	2.889(3)	2.895(5)	2.843(10)	2.782(3)	2.811(2)
**M1−C18**	2.853(5)	2.7890(19)	2.765(3)	2.827(4)	2.880(5)	2.785(9)	2.818(3)	2.829(2)
**M1−C19**	2.797(5)	2.761(2)	2.747(3)	2.738(4)	2.893(5)	2.775(9)	2.794(3)	2.807(2)
**M1−C20**	2.807(5)	2.827(2)	2.799(3)	2.714(4)	2.833(4)	2.843(9)	2.753(3)	2.730(2)
**M1−C21**	2.868(5)	2.847(2)	2.822(3)	2.797(4)	2.813(4)	2.851(9)	2.751(3)	2.756(2)
**N1−M1−Cent1**	101.697	103.383	103.553	101.116	101.091	93.752	93.655	93.761
**N1−M1−Cent2**	100.110	93.478	93.372	103.576	100.480	103.504	101.178	103.628
**N1−M1−Cent3**	96.424	101.405	101.186	93.684	102.385	101.178	103.503	101.236
**Cent1−M1−Cent2**	118.297	118.104	117.980	117.583	114.233	118.287	116.668	117.764
**Cent2−M1−Cent3**	119.515	116.520	116.692	117.626	118.438	117.228	117.408	117.416
**Cent3−M1−Cent1**	114.307	117.436	117.473	116.787	116.037	116.463	117.949	116.684
**M1−Cent$_{avg}$**	**2.578(std. 0.006)**	**2.546(std. 0.011)**	**2.516(std. 0.017)**	**2.498(std. 0.019)**	**2.594(std. 0.012)**	**2.540(std. 0.014)**	**2.524(std. 0.016)**	**2.517(std. 0.016)**
**M1−C$_{avg}$**	**2.843(std. 0.030)**	**2.813(std. 0.034)**	**2.788(std. 0.041)**	**2.771(std. 0.048)**	**2.859(std. 0.038)**	**2.811(std. 0.041)**	**2.795(std. 0.043)**	**2.789(std. 0.047)**
**Cent−M1−Cent avg**	**117.373**	**117.353**	**117.381**	**117.332**	**116.236**	**117.326**	**117.342**	**117.288**

Supplementary Note 4: Solid-State Absorption and Photoluminescence

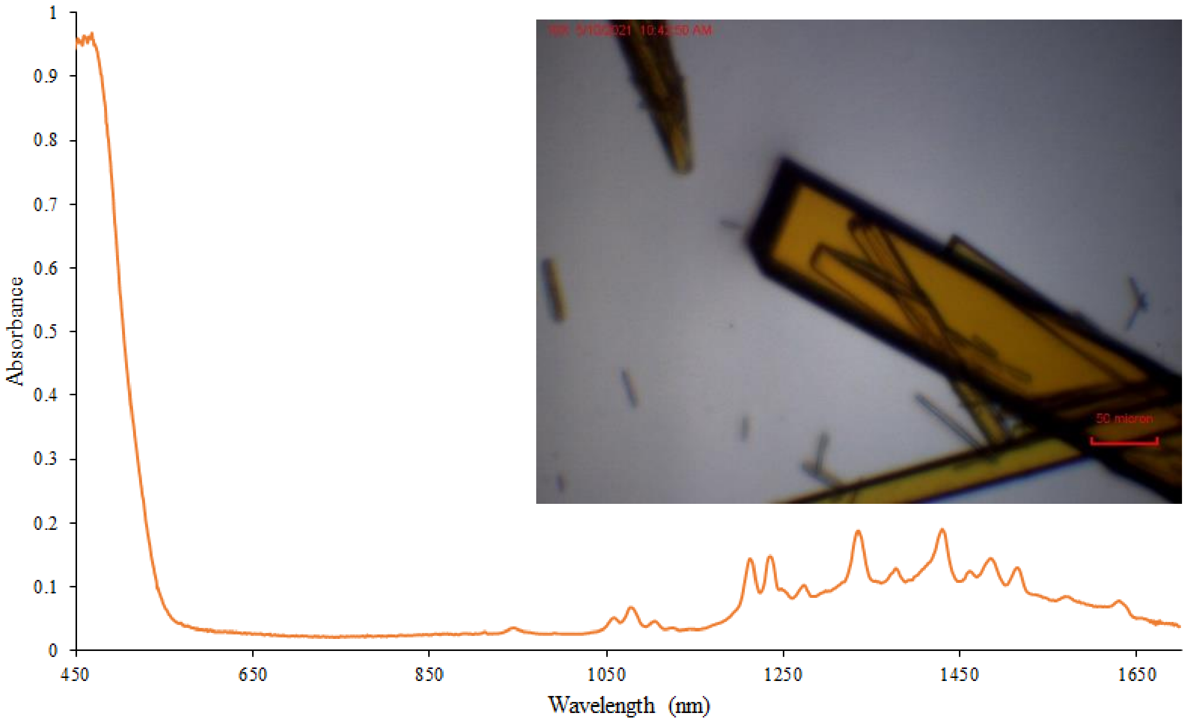

Supplementary Figure 9. **Solid-state absorption spectrum of 1−Sm**. Solid-state absorption spectrum of 1−Sm, collected at room temperature. The crystal used is pictured in the top right. The scale bar represents 50 microns.

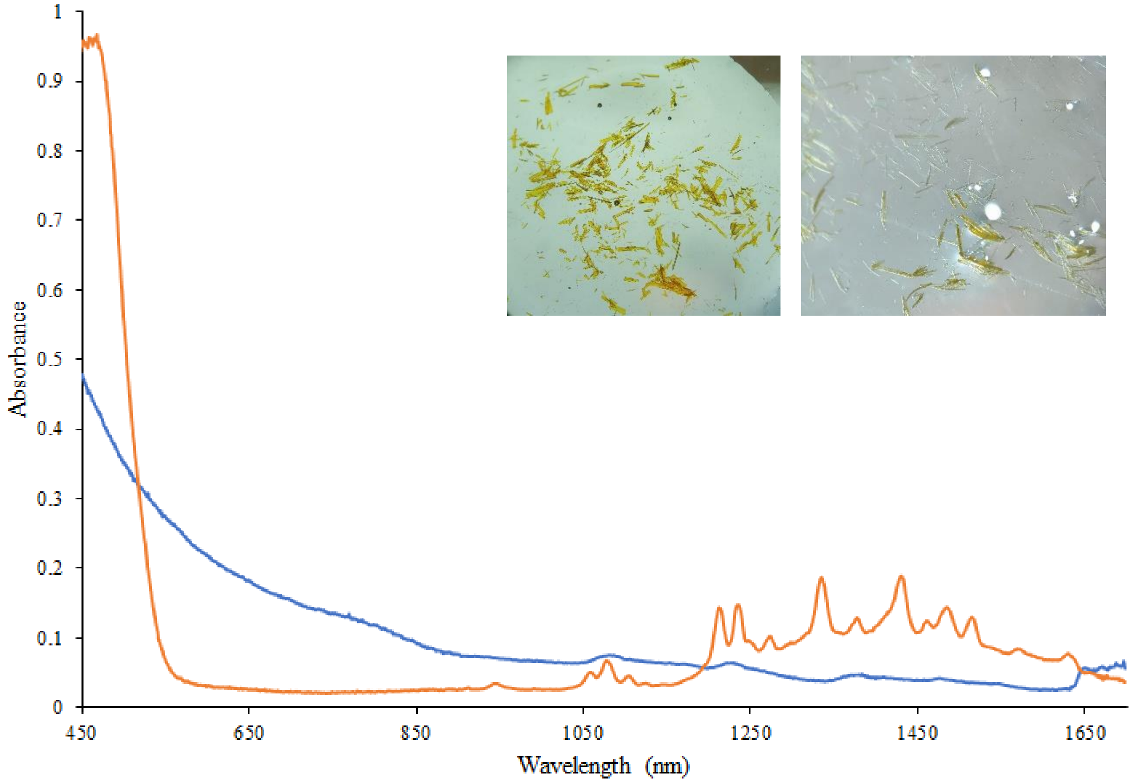

Supplementary Figure 10. **Solid-state absorption spectrum of 1−Sm before and after 24 hours**. Solid-state absorption spectrum of 1−Sm collected using fresh crystals (orange) and after 24 hours of air exposure (blue). The crystal batches used are shown with the fresh batch on the left and after 24 hours of air exposure on the right.

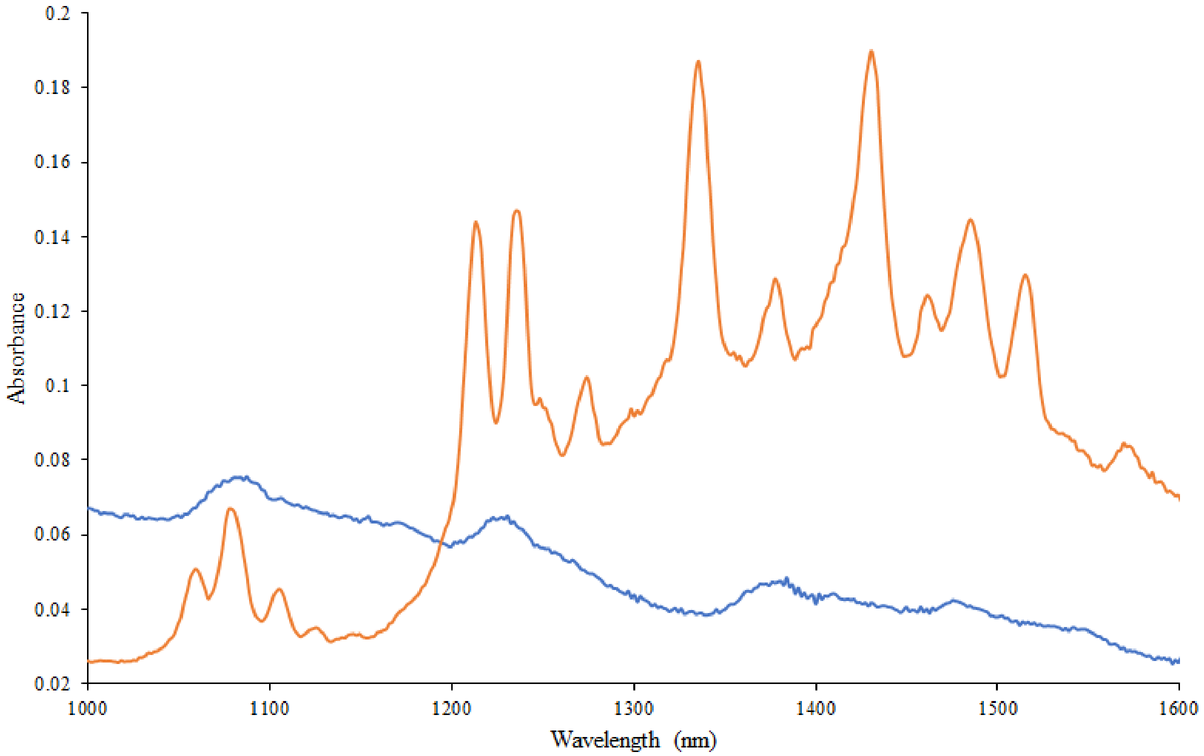

Supplementary Figure 11. **Zoomed solid-state absorption spectrum of 1−Sm before and after 24 hours**. Solid-state absorption spectrum of 1−Sm before (orange) and after (blue) air exposure between 1000 nm and 1600 nm for enhanced clarity of *f*-*f* transitions.

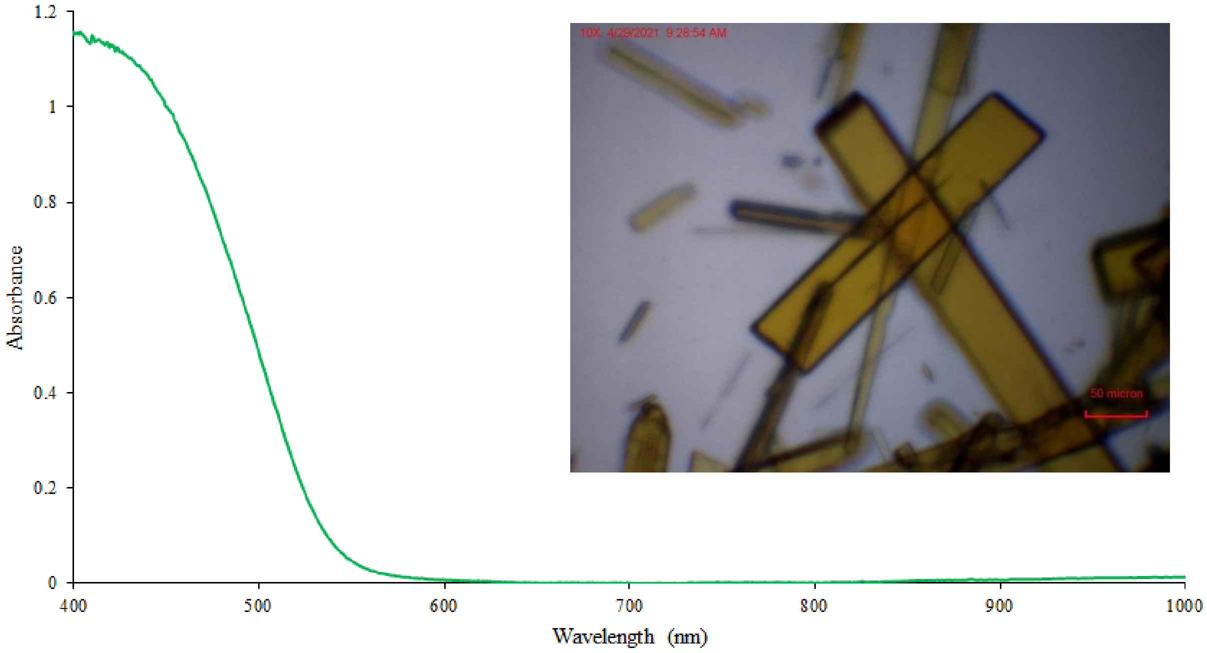

Supplementary Figure 12. **Solid-state absorption spectrum of 1−Gd**. Solid-state absorption spectrum of 1−Gd, collected at room temperature. The crystal used is pictured in the top right. The scale bar represents 50 microns.

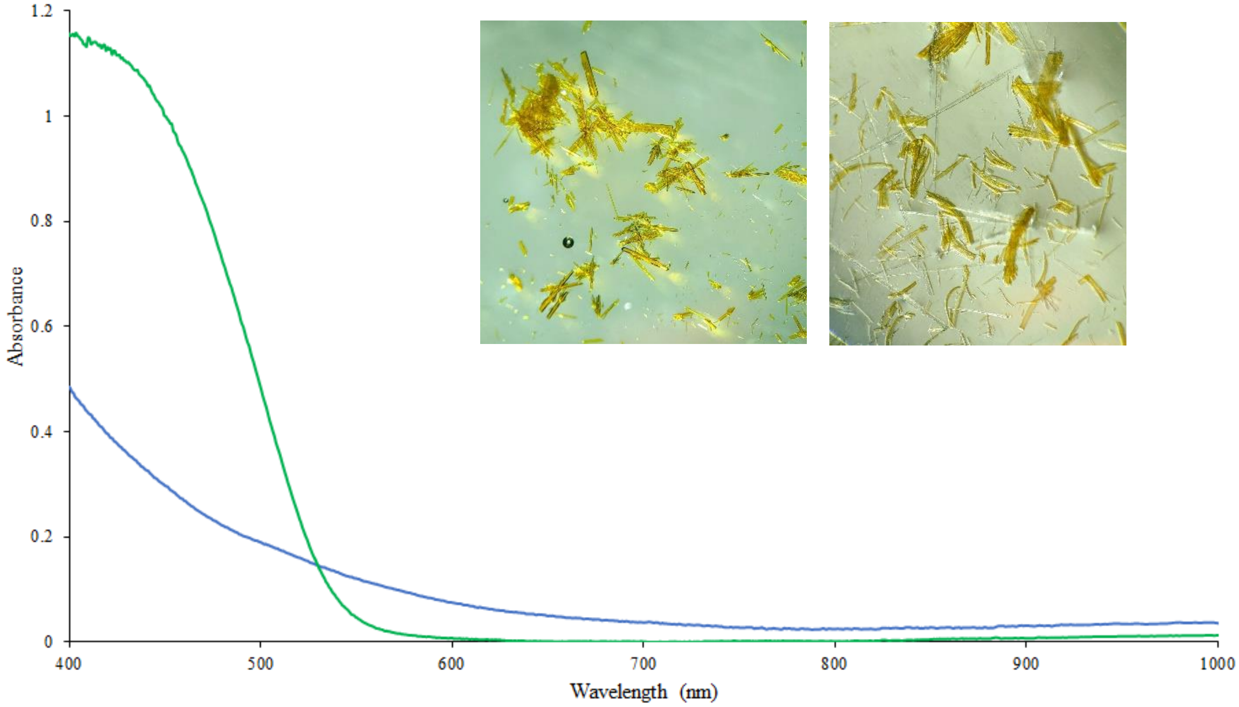

Supplementary Figure 13. **Solid-state absorption spectrum of 1−Gd before and after 24 hours**. Solid-state absorption spectrum of 1−Gd collected using fresh crystals (green) and after 24 hours of air exposure (blue). The crystal batches used are pictured with the fresh crystals on the left and after 24 hours of air exposure on the right.

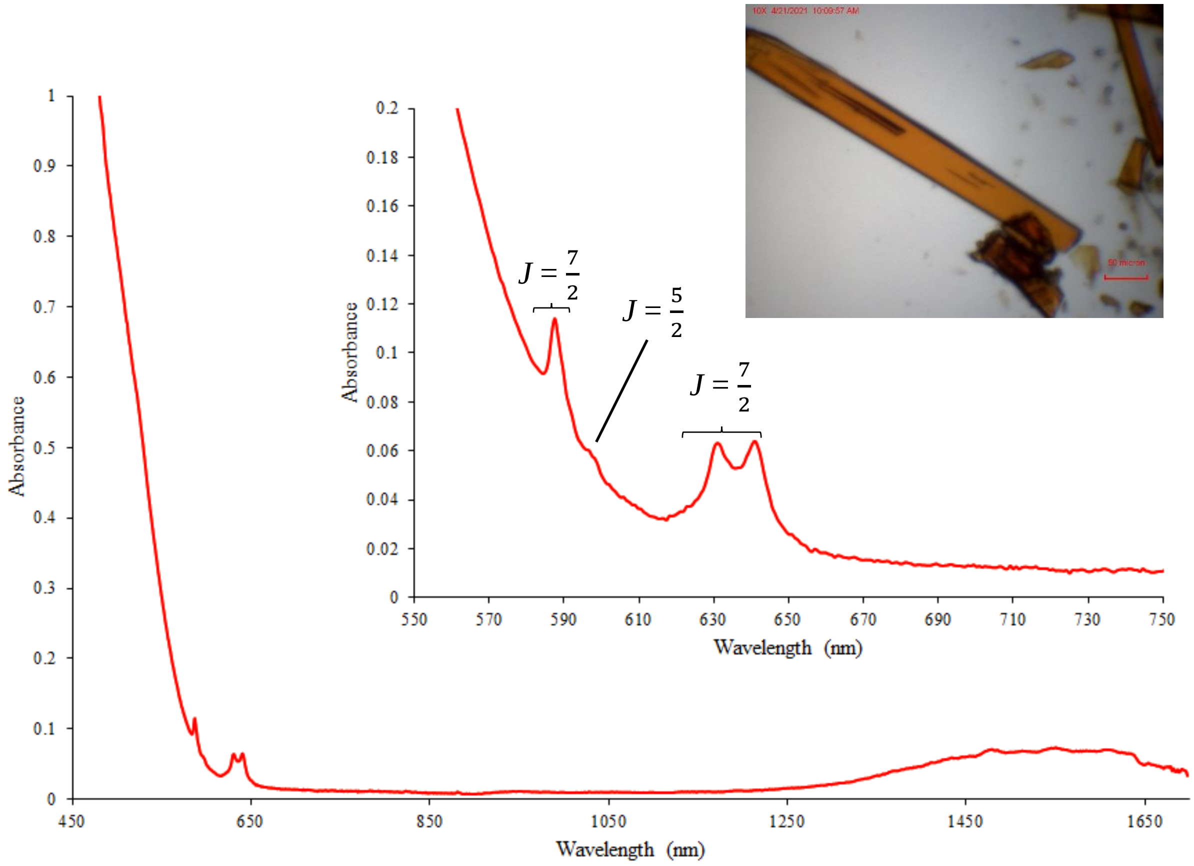

Supplementary Figure 14. **Solid-state absorption spectrum of 1−Cm**. Solid-state absorption spectrum of 1−Cm, collected at room temperature. The crystal used is pictured in the top right. The scale bar represents 50 microns.

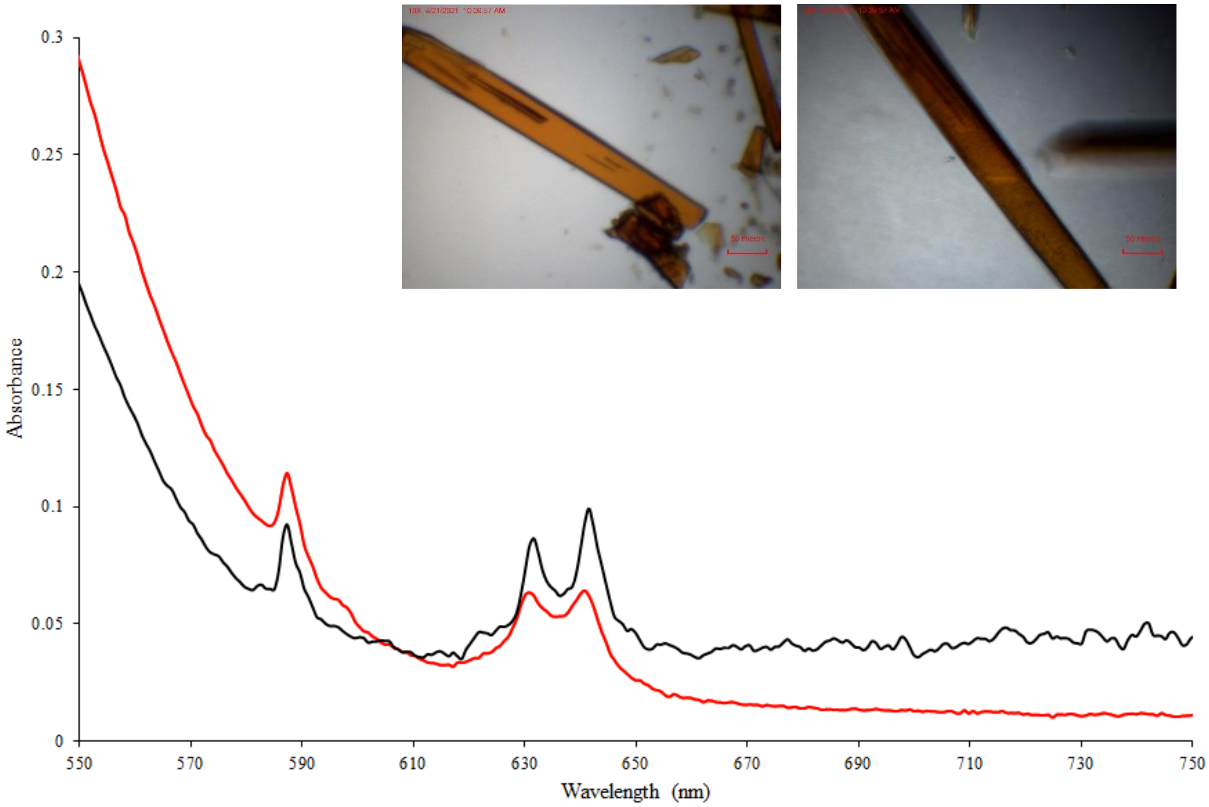

Supplementary Figure 15. **Solid-state absorption spectrum of 1−Cm at low temperature**. Solid-state absorption spectrum of 1−Cm, collected at room temperature (red) compared to −180 °C (black). The crystals used are pictured at room temperature (left) and at −180 °C (right). The scale bar represents 50 microns.

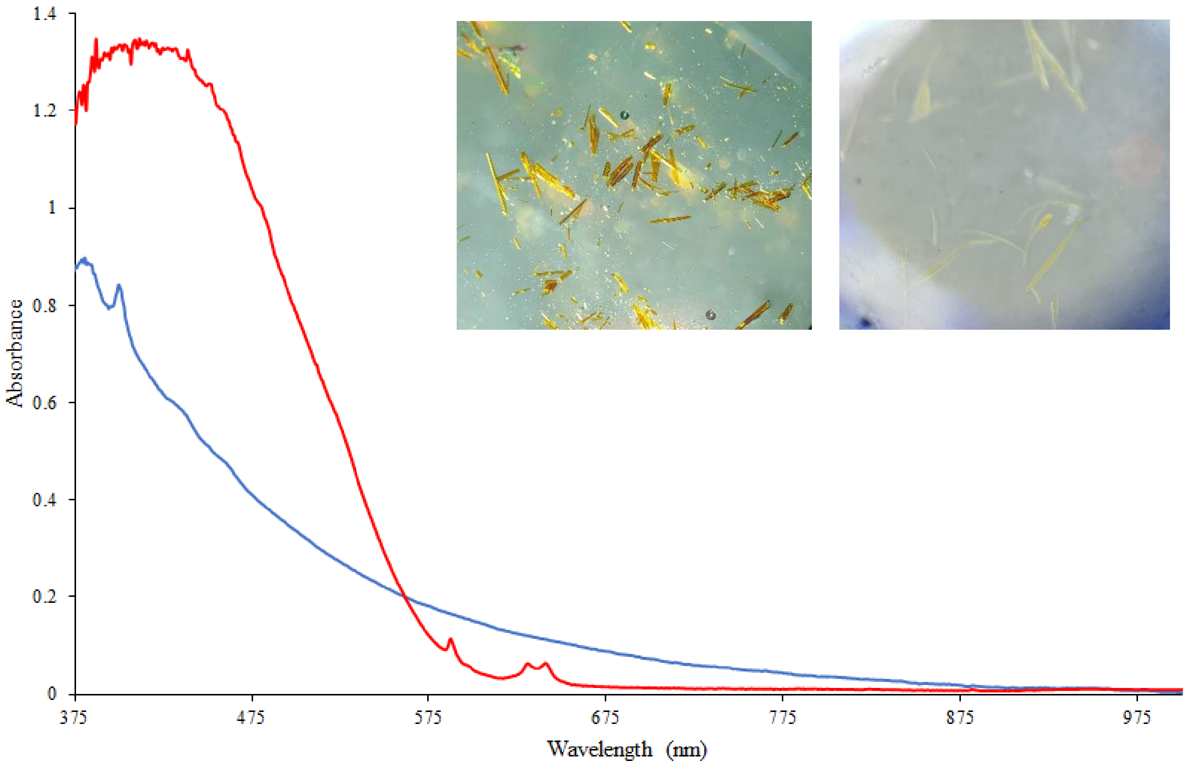

Supplementary Figure 16. **Solid-state absorption spectrum of 1−Cm before and after 24 hours**. Solid-state absorption spectrum of 1−Cm collected using fresh crystals (red) and after 24 hours of air exposure (blue). The crystal batches used are pictured with the fresh crystals on the left and after 24 hours of air exposure on the right.

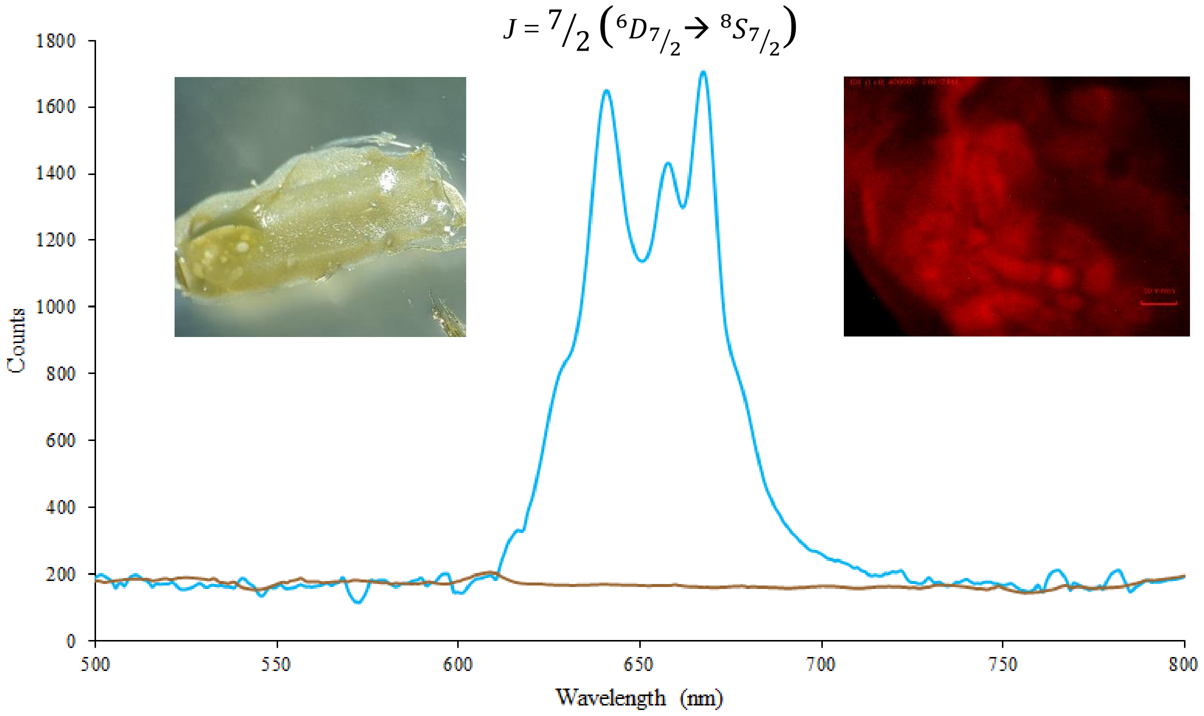

Supplementary Figure 17. **Photoluminescence of 1−Cm and Cp'_3Cm**. Photoluminescence of putative Cp'_3Cm excited at 420 nm. Emission spectra were collected before (blue) and after (brown) 24 hours of air exposure. The scale bar represents 50 microns.

Supplementary Note 5: Solution Phase Absorption Spectra

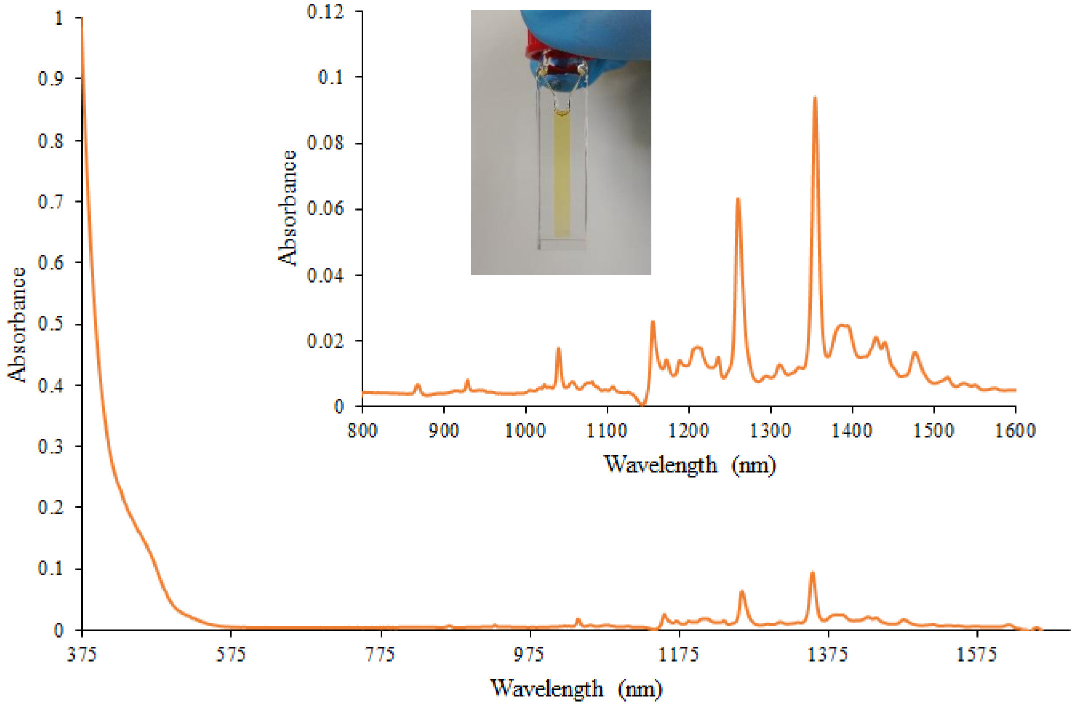

Supplementary Figure 18. **Solution phase absorption spectrum of 1−Sm**. Solution phase absorption spectrum of 1−Sm. A zoomed spectrum is shown in the top right to enhance clarity of *f*-*f* transitions. The sample used is pictured above.

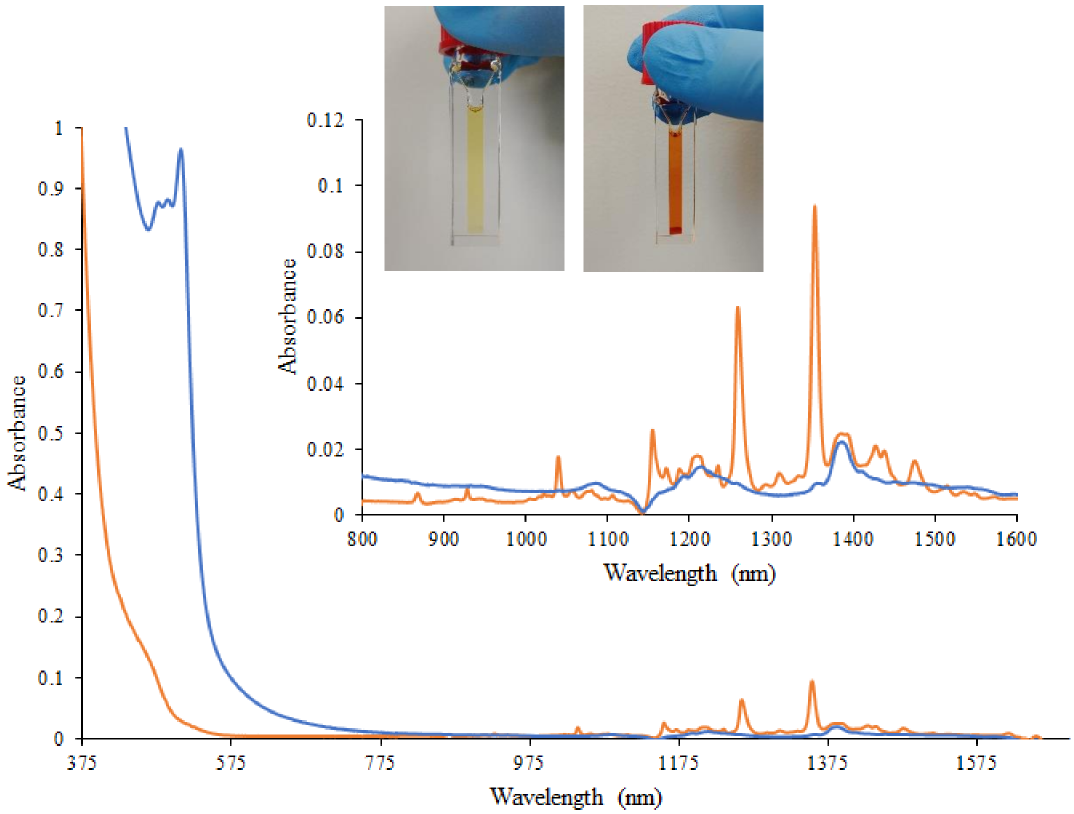

Supplementary Figure 19. **Solution phase absorption spectrum of 1−Sm before and after 24 hours**. Solution phase absorption spectra of 1−Sm. Spectra were collected before (orange) and after (blue) 24 hours of air exposure. The sample used is pictured above before (left) and after (right) 24 hours of air exposure. A zoomed spectrum is provided to enhance clarity of the *f*-*f* transitions.

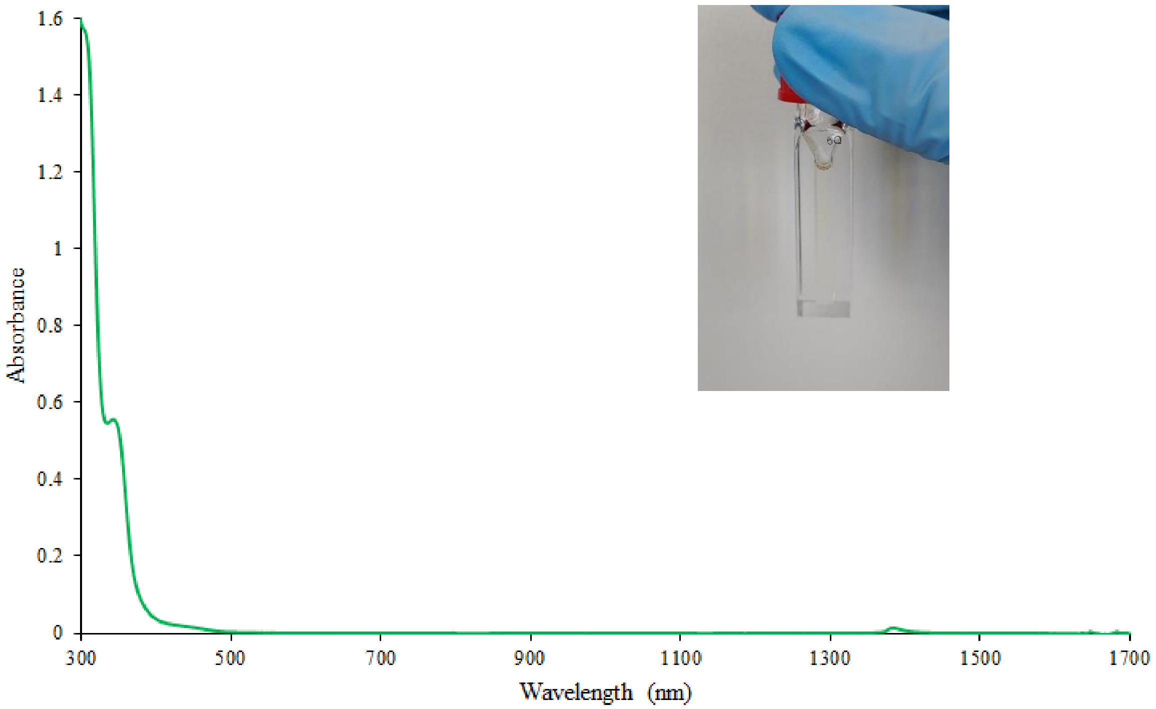

Supplementary Figure 20. **Solution phase absorption spectrum of 1−Gd**. Solution phase absorption spectrum of 1−Gd. The sample used is pictured above.

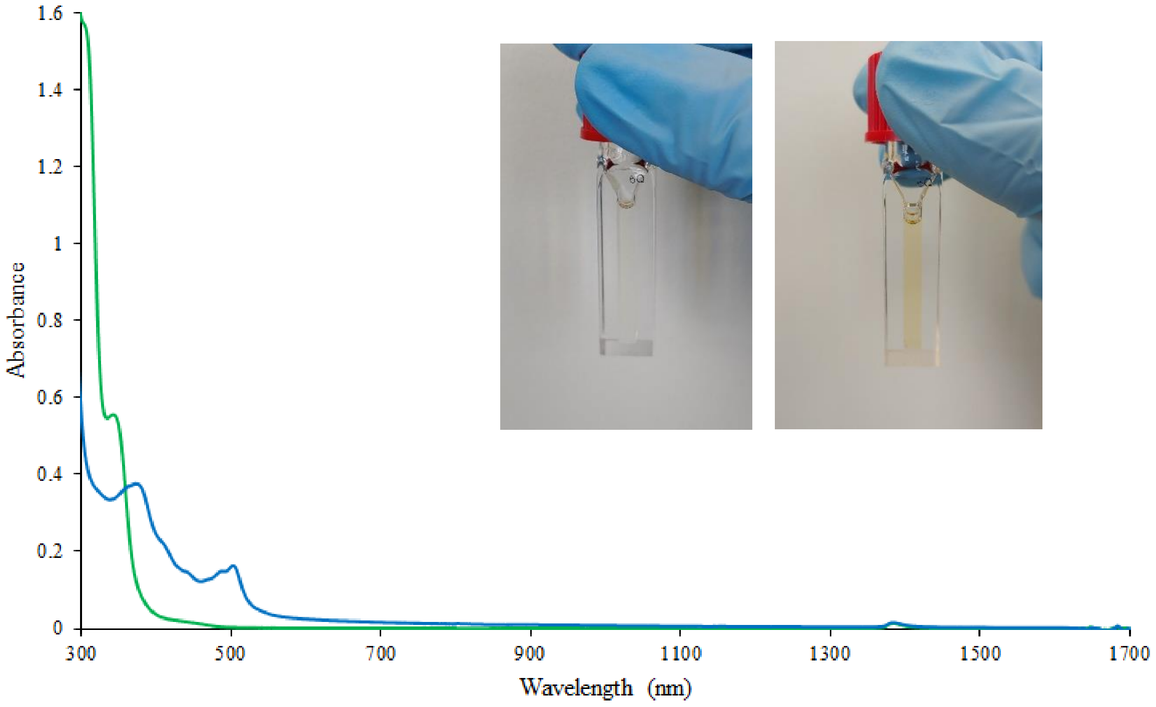

Supplementary Figure 21. **Solution phase absorption spectrum of 1−Sm before and after 24 hours**. Solution phase absorption spectra of 1−Gd. Spectra were collected before (green) and after (blue) 24 hours of air exposure. The sample used is pictured above before (left) and after (right) 24 hours of air exposure.

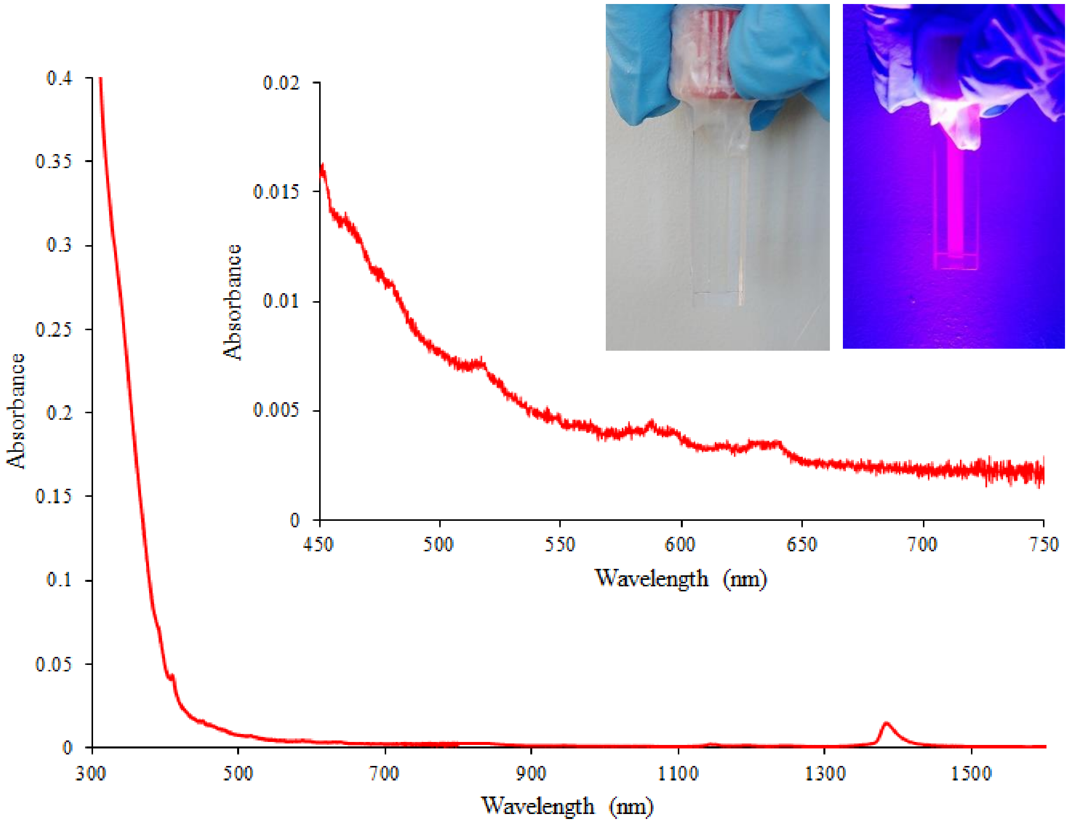

Supplementary Figure 22. **Solution phase absorption spectrum of 1−Cm**. Solution phase absorption spectrum of 1−Cm. A zoomed spectrum is shown to view the very weak *f*-*f* transitions of Cm^{3+}. The sample used is pictured in the top right and again under a blacklight.

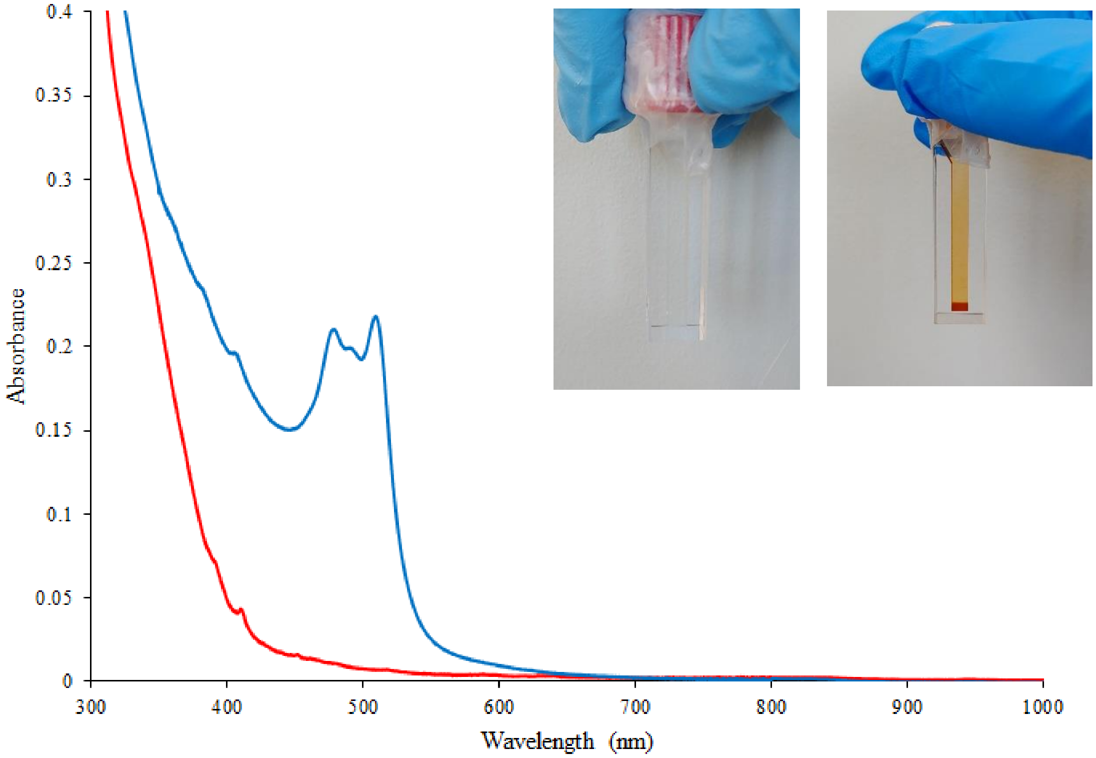

Supplementary Figure 23. **Solution phase absorption spectrum of 1−Cm before and after 24 hours**. Solution phase absorption spectra of 1−Cm. Spectra were collected before (red) and after (blue) 24 hours of air exposure. The sample used is pictured above before (left) and after (right) 24 hours of air exposure.

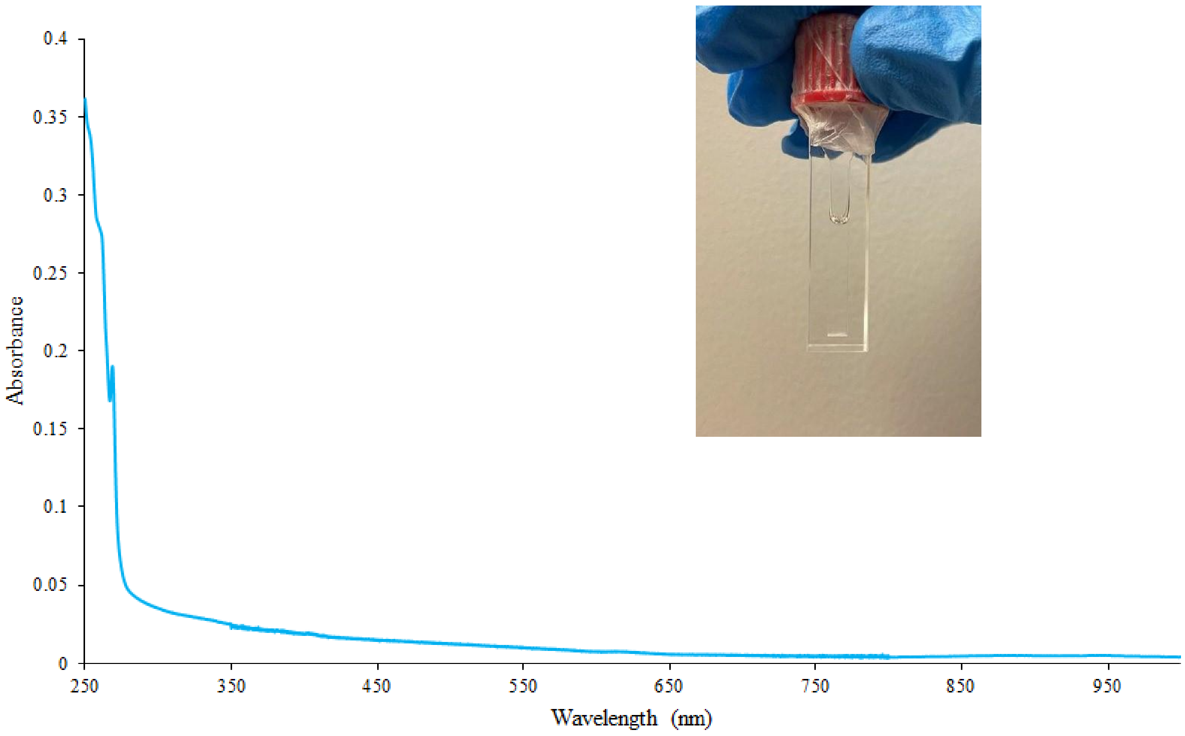

Supplementary Figure 24. **Solution phase absorption spectrum of Cp'_3Cm**. Solution phase absorption spectrum of a putative Cp'_3Cm solution. The solution used is pictured.

Supplementary Note 6: Computational Details

To get insight on the electronic structure, spectroscopic properties, and bonding in 1−Sm, 1−Gd, 1−Cm and $Cp_3'Cm$, the *ab-initio* complete active space self-consistent field (CASSCF) along time-dependent DFT (TDDFT) approximations were employed. [7] Details of each calculation are described below.

Theoretical Models and Geometry Optimizations

The structures of 1−Sm, 1−Gd, and 1−Cm were obtained from the crystallographic information file (CIF) and were used as input for the geometry optimizations. Since no crystallographic structures were obtained for Cp'_3Cm, a geometry optimization on a hypothetical system was carried out. All these calculations were performed in ADF2019[8] using the generalized gradient approximation (GGA) Perdew-Burke-Ernzerhof (PBE) in conjunction with the Slater-type basis functions (STO) triple-zeta potential (TZP). [9] The scalar relativistic (SR) effects were incorporated via the zeroth-order relativistic approximation (ZORA). [10] Due to 1−Sm, 1−Gd, and 1−Cm are bimetallic systems, their size represents a limitation from the theoretical perspective. Therefore, a model consisting of one Cp'_3M unit (M = Sm, Gd, Cm) coordinated to pyridine was employed.

CASSCF Calculations

The starting wavefunctions were calculated at Hartree-Fock (HF) level of theory along with the all-electron ANO-RCC Gaussian-type basis sets contracted to TZP quality. [11,12] The second-order Douglas-Kroll-Hess (DKH2) Hamiltonian was employed to account for the scalar relativistic effects. [13] This resulting wavefunction was subjected to state-average (SA) CASSCF calculations to mostly recover the static correlation. Two active spaces were calculated: 1) n electrons in seven f orbitals, CAS(n, 7) with n = 5,7,7 for Sm, Gd and Cm, respectively 2) an extension of the first one that includes one bonding ligand orbital and two extra electrons, that is a CAS(n + 2, 8). A third active space including 6 extra electrons and 6 extra orbitals corresponding to the bonding and antibonding $6d_{z^2}$, $6d_{xz}$ and $6d_{yz}$ orbitals was attempted, however it became impractical. To account

for the dynamic correlation effects, the multiconfigurational pair-density functional theory (MC-pDFT) was employed using tPBE on-top functional. [14] The spin-orbit (SO) coupling was included by state interactions between the CASSCF wavefunctions using the restricted active space state interaction (RASSI) method. [15] All these calculations were performed in OpenMolcas. [16]

Bonding Analyses

Natural Bond Orbital (NBO) analyses[17] were performed with the NBO software implemented in the Amsterdam Density Functional (ADF) suite within AMS2019. [18] The fully optimized structures were used to calculate the Natural Localized Molecular Orbitals (NLMOs) of 1−Sm, 1−Gd, 1−Cm and Cp'_3Cm. The level of theory used was similar to those used for the geometry optimizations except that the functional used was the hybrid GGA PBE0. Given that the NBO formalism produces similar results independent of the basis set or whether the density comes from DFT or wavefunction-based calculations, we considered that calculating the whole experimental molecule would be best to be analyzed. Conversely, the topology of the electron density is considerably more sensitive to the origin of the molecular electron density, thus we used the CASSCF ground-state wavefunction to obtain the molecular electron density to be analyzed through QTAIM calculations with MultiWFN. [19]

Supplementary Note 7: Additional Computational Results

Supplementary Table 5. Experimental and calculated average M–(Cp′ cent) distances (Å) and angles (degrees) in 1−Sm, 1−Gd and 1−Cm. The effect of coordinating the 4,4′–bipyridine is also observed in the calculated bond distances of Cp'_3M (M=Sm, Gd, Cm) systems shown in Supplementary Table 6. As observed below, bond lengths in Cp'_3M are smaller than in 1−M due to the reduced coordination.

	Experimental			Calculated		
	**1−Sm**	**1−Gd**	**1−Cm**	**1−Sm**	**1−Gd**	**1−Cm**
Avg M–(Cp′ cnt)	2.516	2.498	2.517	2.546	2.516	2.541
$M–N_{Bpy}$	2.626	2.592	2.596	2.729	2.660	2.670
Avg Cnt–M–Cnt	117.4	117.3	117.3	117.8	117.6	117.5
Avg Cnt–M–N	99.6	99.5	99.5	98.2	99.0	99.1

Supplementary Table 6. Experimental and calculated average M – (Cp′ cent) distances (Å) in Cp'_3M (M = Sm, Gd, Cm). Cp'_3Sm and Cp'_3Gd systems were optimized for comparative purposes using the same level of theory described in the computational details.

	Experimental			Calculated		
	**Cp'_3Sm[a]**	**Cp'_3Gd[b]**	**Cp'_3Cm**	**Cp'_3Sm**	**Cp'_3Gd**	**Cp'_3Cm**
Avg M–(Cp′ cnt)	2.461	2.437	-	2.475	2.444	2.472

[a] *J. Am. Chem. Soc.* 2015, 137, 1, 369–382 ([2])
[b] *J. Am. Chem. Soc.* 2013, 135, 26, 9857–9868 ([3])

Supplementary Table 7. Theoretical assignment of the experimental absorption bands observed in 1−Sm. The assignment was done based on the calculated position of the SO-pDFT states shown in **Supplementary Table 8**.

**Experimental Bands**		**Assignment**
**Wavelength (nm)**	**Wavenumber (cm^{-1})**	
1575	6349.2	5/2(^{6}H) → 15/2 (^{6}H)<br>5/2(^{6}H) → 1/2 (^{6}F)
1518	6587.6	5/2(^{6}H) → 15/2 (^{6}H)<br>5/2(^{6}H) → 1/2 (^{6}F)
1475	6779.7	5/2(^{6}H) → 15/2 (^{6}H)<br>5/2(^{6}H) → 1/2 (^{6}F)
1460	6849.3	5/2(^{6}H) → 15/2 (^{6}H)<br>5/2(^{6}H) → 1/2 (^{6}F)
1430	6993.0	5/2(^{6}H) → 3/2 (^{6}F)
1375	7272.7	5/2(^{6}H) → 5/2 (^{6}F)
1235	8097.2	5/2(^{6}H) → 7/2 (^{6}F)
1215	8230.5	5/2(^{6}H) → 7/2 (^{6}F)
1115	8968.6	5/2(^{6}H) → 9/2 (^{6}F)
1075	9302.3	5/2(^{6}H) → 9/2 (^{6}F)
1060	9434.0	5/2(^{6}H) → 9/2 (^{6}F)

Supplementary Table 8. Assignment of the SO−pDFT states for 1−Sm in terms of the total angular momentum quantum number, J. The most predominant Russell-Saunders term is also indicated in the second column. The energies and wavelenghts correspond to the Kramers components of the different manifolds.

**Manifold**		**Position of SO-pDFT states**	
**J**	**Spectroscopic Term**	**Energy (cm^{-1})**	**Wavelength (nm)**
5/2	^{6}H	0<br>58.4725<br>223.419	-<br>171020.6<br>44759
7/2	^{6}H	728.9291	13718.8

		1066.28<br>1197.14<br>1217.94	9378.4<br>8353.2<br>8210.6
9/2	^{6}H	2175.682<br>2302.326<br>2408.72<br>2434.236<br>2450.924	4596.3<br>4343.4<br>4151.6<br>4108.1<br>4080.1
11/2	^{6}H	3362.499<br>3483.949<br>3593.22<br>3785.206<br>3817.926<br>3854.888	2974.0<br>2870.3<br>2783.0<br>2641.9<br>2619.2<br>2594.1
13/2	^{6}H	4884.269<br>5043.233<br>5203.371<br>5278.985<br>5338.677<br>5340.178<br>5354.613	2047.4<br>1982.9<br>1921.8<br>1894.3<br>1873.1<br>1872.6<br>1867.5
15/2 + 1/2	^{6}H + ^{6}F	6305.665<br>6364.099<br>6486.014<br>6532.931<br>6574.991<br>6642.288<br>6702.247<br>6743.443<br>6861.831	1585.9<br>1571.3<br>1541.8<br>1530.7<br>1520.9<br>1505.5<br>1492.0<br>1482.9<br>1457.3
3/2	^{6}F	6876.426<br>6995.769	1454.2<br>1429.4
5/2	^{6}F	7290.769<br>7361.329<br>7371.416	1371.6<br>1358.5<br>1356.6
7/2	^{6}F	8206.31<br>8215.612<br>8216.662<br>8292.328	1218.6<br>1217.2<br>1217.0<br>1205.9
9/2	^{6}F	9378.206<br>9420.438<br>9438.025<br>9562.972<br>9597.42	1066.3<br>1061.5<br>1059.5<br>1045.7<br>1041.9

11/2	^{6}F	10836.26<br>10852.76<br>10893.39<br>10980.48<br>11095.79<br>11150.84	922.8<br>921.4<br>918.0<br>910.7<br>901.2<br>896.8

Supplementary Table 9. Theoretical assignment of predicted absorption bands in 1−Gd. The assignment was done based on the calculated position of the SO-pDFT states shown in **Supplementary Table 10**.

Predicted Bands		Assignment
Wavelength (nm)	Wavenumber (cm^{-1})	
334	29967	7/2(^{8}S) → 7/2 (^{6}D)
329	30360	7/2(^{8}S) → 5/2 (^{6}D)

Supplementary Table 10. Assignment of the SO−pDFT states for 1−Gd in terms of the total angular momentum quantum number, J. The most predominant Russell-Saunders term is also indicated in the second column. The energies and wavelenghts correspond to the Kramers components of the different manifolds.

Manifold		Position of SO-pDFT states	
J	Spectroscopic Term	Energy (cm^{-1})	Wavelength (nm)
7/2	^{8}S	0<br>2.2334<br>6.7497<br>13.6279	-<br>4477478.3<br>1481547.3<br>733788.8
7/2	^{6}D	29344<br>29953.06<br>29965.58<br>30059.75	340.8<br>333.9<br>333.7<br>332.7
5/2	^{6}D	30345.8<br>30360.33<br>30372.94	329.5<br>329.4<br>329.2

Supplementary Table 11. Theoretical assignment of the experimental absorption bands observed in 1−Cm. The assignment was done based on the calculated position of the SO-pDFT states shown in **Supplementary Table 12**.

**Experimental Bands**		**Assignment**
**Wavelength (nm)**	**Wavenumber (cm^{-1})**	
630	15873	$7/2(^8S) \rightarrow 7/2\ (^6D)$
650	15384	$7/2(^8S) \rightarrow 7/2\ (^6D)$

Supplementary Table 12. Assignment of the SO−pDFT states for 1−Cm in terms of the total angular momentum quantum number, J. The most predominant Russell-Saunders term is also indicated in the second column. The energies and wavelenghts correspond to the Kramers components of the different manifolds.

**Manifold**		**Position of SO-pDFT states**	
**J**	**Spectroscopic Term**	**Energy (cm^{-1})**	**Wavelength (nm)**
7/2	^8S	0<br>40.2677<br>187.123<br>385.236	-<br>248338.0<br>53440.8<br>25958.1
7/2	^6D	15216.6<br>15266.9<br>15353.4<br>16087.8	657.2<br>655.0<br>651.3<br>621.6
5/2	^6D	16650.1<br>16657.7<br>16735.9	600.6<br>600.3<br>597.5

Supplementary Table 13. Theoretical assignment of the experimental emission bands observed in Cp'_3Cm. The assignment was done based on the calculated position of the SO-pDFT states shown in **Supplementary Table 14**.

Experimental Bands		Assignment
**Wavelength (nm)**	**Wavenumber (cm^{-1})**	
645	15504	7/2 (^6D) → 7/2(^8S)
660	15152	7/2 (^6D) → 7/2(^8S)
670	14925	7/2 (^6D) → 7/2(^8S)

Supplementary Table 14. Assignment of the SO–pDFT states for Cp'_3Cm in terms of the total angular momentum quantum number, J. The most predominant Russell-Saunders term is also indicated in the second column. The energies and wavelenghts correspond to the Kramers components of the different manifolds.

Manifold		Position of SO-pDFT states	
**J**	**Spectroscopic Term**	**Energy (cm^{-1})**	**Wavelength (nm)**
7/2	^8S	0<br>25.333<br>129.187<br>351.218	-<br>394742.0<br>77407.1<br>28472.4
7/2	^6D	15121.4<br>15408.3<br>15448.1<br>15698.7	661.3<br>649.0<br>647.3<br>637.0
5/2	^6D	16365.6<br>16401.2<br>16559.1	611.0<br>609.7<br>603.9

Supplementary Table 15. Selected Natural Localized Molecular Orbitals (NLMOs) and their hybrid compositions describing the metal-ligand bonds of 1−Sm, 1−Gd, 1−Cm, and Cp'_3Cm. NLMOs were obtained from PBE/STO-TZP calculations.

		**NLMO Hybrid composition**	
		α	β
M−Cp′(1)	**1−Sm**	14% Sm(0.25 5d; 0.75 4f)	4% Sm(0.79 5d; 0.21 4f)
		85% C(1.00 2p)	95% C(1.00 2p)
	**1−Gd**	6% Gd(0.01 6s; 0.98 5d)	6% Gd(0.70 5d; 0.29 4f)
		93% C(1.00 2p)	93% C(1.00 2p)
	**1−Cm**	9% Cm(0.01 7s; 0.99 6d)	9% Cm(0.01 7s; 0.64 6d; 0.35 5f)
		90% C(0.02 2s; 0.98 2p)	90% C(1.00 2p)
	Cp'_3Cm	9% Cm(0.02 7s; 0.98 6d)	8% Cm(0.81 6d; 0.18 5f)
		90% C(0.01 2s; 0.99 2p)	91% C(1.00 2p)
M−Cp′(2)	**1−Sm**	16% Sm(0.19 5d; 0.81 4f)	4% Sm(0.85 5d; 0.14 4f)
		83% C(1.00 2p)	94% C(1.00 2p)
	**1−Gd**	4% Gd(0.01 6s; 0.98 5d)	6% Gd(0.62 5d; 0.37 4f)
		94% C(1.00 2p)	93% C(1.00 2p)
	**1−Cm**	9% Cm(0.01 7s; 0.99 6d)	9% Cm(0.01 7s; 0.54 6d; 0.45 5f)
		90% C(0.02 2s; 0.98 2p)	90% C(1.00 2p)
	Cp'_3Cm	8% Cm(0.02 7s; 0.98 6d)	8% Cm(0.82 6d; 0.17 5f)
		91% C(0.01 2s; 0.99 2p)	90% C(0.01 2s; 0.99 2p)
M−Cp′(3)	**1−Sm**	10% Sm(0.35 5d; 0.65 4f)	4% Sm(0.74 5d; 0.25 4f)
		88% C(1.00 2p)	95% C(1.00 2p)
	**1−Gd**	6% Gd(0.01 6s; 0.98 5d)	6% Gd(0.79 5d; 0.20 4f)
		93% C(1.00 2p)	93% C(1.00 2p)
	**1−Cm**	9% Cm(0.01 7s; 0.99 6d)	9% Cm(0.01 7s; 0.72 6d; 0.28 5f)
		90% C(0.02 2s; 0.98 2p)	90% C(1.00 2p)
	Cp'_3Cm	9% Cm(0.01 7s; 0.99 6d)	7% Cm(0.80 6d; 0.19 5f)
		90% C(0.01 2s; 0.99 2p)	91% C(0.01 2s; 0.99 2p)
M−N	**1−Sm**	4% Sm(0.17 6s; 0.68 5d; 0.15 4f)	4% Sm(0.19 6s; 0.73 5d; 0.08 4f)
		93% N(0.32 2s; 0.68 2p)	93% N(0.32 2s; 0.68 2p)
	**1−Gd**	4% Gd(0.20 6s; 0.80 5d)	4% Gd(0.19 6s; 0.73 5d; 0.08 4f)
		92% N(0.31 2s; 0.69 2p)	93% N(0.32 2s; 0.68 2p)
	**1−Cm**	7% Cm(0.13 7s; 0.87 6d)	6% Cm(0.11 7s; 0.72 6d; 0.16 5f)
		90% N(0.30 2s; 0.70 2p)	90% N(0.30 2s; 0.70 2p)

OPTIMIZED MOLECULAR GEOMETRIES

1−Sm

Sm	4.73061123	-2.64827356	3.23460013
Si	6.32535635	-3.97467007	6.92868079
Si	5.46783406	1.71748016	3.65914492
Si	1.88048446	-5.84812361	2.44997454
N	2.58751183	-1.59397196	1.91447343
C	4.83599384	-1.67571701	5.83810336
H	5.62082524	-0.95297599	6.04547444
C	3.53587486	-1.35620278	5.37143660
H	3.16545771	-0.35306530	5.16661526
C	4.23674051	-4.58891425	1.20455271
H	3.71680367	-4.30472419	0.29027430
C	4.68984839	-5.45576020	3.26576506
H	4.58017983	-5.93344087	4.23576223
C	3.61693761	-5.14045162	2.37013464
C	4.93915384	-3.08777958	6.02092169
C	1.55383840	-0.95953170	2.50035640
H	1.54823428	-0.95098625	3.58990569
C	3.65960257	-3.62171586	5.67165078
H	3.37371423	-4.66845425	5.73096225
C	2.80644318	-2.56965708	5.26726061
H	1.76568268	-2.68376220	4.96663824
C	0.53167318	-0.32917932	1.79855273
H	-0.25448173	0.16076514	2.37140448
C	5.92041735	-5.09549452	2.67229726
H	6.91056485	-5.23076230	3.10689776
C	6.30444126	-1.47620526	1.24708588
H	6.28504236	-1.96485097	0.27527354
C	5.63953617	-4.57170528	1.38317315

H	6.37771499	-4.24585551	0.65455388
C	5.50950065	-0.37941290	1.64196932
H	4.77236554	0.12002161	1.01528940
C	5.86700375	0.01837363	2.96573243
C	0.53295146	-0.33061104	0.39508316
C	6.91088248	-0.87861888	3.37418966
H	7.44542333	-0.84124070	4.32192948
C	2.59231655	-1.59436932	0.56604548
H	3.43719222	-2.09595583	0.09150143
C	7.17805939	-1.78944106	2.32888931
H	7.93487617	-2.57280314	2.33576119
C	1.61207300	-0.99527739	-0.21404364
H	1.71161174	-1.05389461	-1.29704516
C	5.74114225	1.85009753	5.52357790
H	5.01138576	1.26869555	6.10411461
H	5.64958239	2.90306964	5.83231463
H	6.75041334	1.51498594	5.80770322
C	8.01700921	-3.39675309	6.31058630
H	8.16325316	-3.62470261	5.24433964
H	8.82284561	-3.89221468	6.87386272
H	8.14339774	-2.31161519	6.44288380
C	6.19039511	-3.56237198	8.76857895
H	6.27276188	-2.47825664	8.93708372
H	6.98549909	-4.05776197	9.34726117
H	5.22091824	-3.88858067	9.17370460
C	6.62187740	2.95745679	2.81603453
H	7.67475966	2.71734845	3.02688822
H	6.42987796	3.98328680	3.16699725
H	6.49106614	2.93956620	1.72383536
C	3.68530103	2.20714105	3.24626093
H	3.48594231	2.12399444	2.16712251

H	3.49692504	3.25183299	3.53745906
H	2.95383104	1.57675820	3.77249227
C	1.37023903	-6.23408378	4.22758993
H	2.10261670	-6.88977031	4.72211638
H	0.40383084	-6.76136193	4.23449532
H	1.26009752	-5.32580088	4.83706170
C	1.87194306	-7.46387598	1.46547441
H	2.15628556	-7.28813541	0.41721176
H	0.87639399	-7.93450834	1.47455648
H	2.59166916	-8.18127972	1.88719776
C	0.60721817	-4.68287054	1.67459018
H	0.50480912	-3.75082793	2.24881153
H	-0.38001366	-5.16893308	1.63919125
H	0.88039169	-4.41628902	0.64224100
C	6.18825932	-5.84722154	6.71945056
H	5.18935419	-6.21408461	7.00014424
H	6.91730934	-6.35140539	7.37210919
H	6.39176104	-6.16554499	5.68706264
N	-2.58751183	1.59397196	-1.91447343
C	-1.55383840	0.95953170	-2.50035640
H	-1.54823428	0.95098625	-3.58990569
C	-0.53167318	0.32917932	-1.79855273
H	0.25448173	-0.16076514	-2.37140448
C	-0.53295146	0.33061104	-0.39508316
C	-2.59231655	1.59436932	-0.56604548
H	-3.43719222	2.09595583	-0.09150143
C	-1.61207300	0.99527739	0.21404364
H	-1.71161174	1.05389461	1.29704516
Sm	-4.73061123	2.64827356	-3.23460013
Si	-6.32535635	3.97467007	-6.92868079
Si	-5.46783406	-1.71748016	-3.65914492

Si	-1.88048446	5.84812361	-2.44997454
C	-4.83599384	1.67571701	-5.83810336
H	-5.62082524	0.95297599	-6.04547444
C	-3.53587486	1.35620278	-5.37143660
H	-3.16545771	0.35306530	-5.16661526
C	-4.23674051	4.58891425	-1.20455271
H	-3.71680367	4.30472419	-0.29027430
C	-4.68984839	5.45576020	-3.26576506
H	-4.58017983	5.93344087	-4.23576223
C	-3.61693761	5.14045162	-2.37013464
C	-4.93915384	3.08777958	-6.02092169
C	-3.65960257	3.62171586	-5.67165078
H	-3.37371423	4.66845425	-5.73096225
C	-2.80644318	2.56965708	-5.26726061
H	-1.76568268	2.68376220	-4.96663824
C	-5.92041735	5.09549452	-2.67229726
H	-6.91056485	5.23076230	-3.10689776
C	-6.30444126	1.47620526	-1.24708588
H	-6.28504236	1.96485097	-0.27527354
C	-5.63953617	4.57170528	-1.38317315
H	-6.37771499	4.24585551	-0.65455388
C	-5.50950065	0.37941290	-1.64196932
H	-4.77236554	-0.12002161	-1.01528940
C	-5.86700375	-0.01837363	-2.96573243
C	-6.91088248	0.87861888	-3.37418966
H	-7.44542333	0.84124070	-4.32192948
C	-7.17805939	1.78944106	-2.32888931
H	-7.93487617	2.57280314	-2.33576119
C	-5.74114225	-1.85009753	-5.52357790
H	-5.01138576	-1.26869555	-6.10411461
H	-5.64958239	-2.90306964	-5.83231463

H	-6.75041334	-1.51498594	-5.80770322
C	-8.01700921	3.39675309	-6.31058630
H	-8.16325316	3.62470261	-5.24433964
H	-8.82284561	3.89221468	-6.87386272
H	-8.14339774	2.31161519	-6.44288380
C	-6.19039511	3.56237198	-8.76857895
H	-6.27276188	2.47825664	-8.93708372
H	-6.98549909	4.05776197	-9.34726117
H	-5.22091824	3.88858067	-9.17370460
C	-6.62187740	-2.95745679	-2.81603453
H	-7.67475966	-2.71734845	-3.02688822
H	-6.42987796	-3.98328680	-3.16699725
H	-6.49106614	-2.93956620	-1.72383536
C	-3.68530103	-2.20714105	-3.24626093
H	-3.48594231	-2.12399444	-2.16712251
H	-3.49692504	-3.25183299	-3.53745906
H	-2.95383104	-1.57675820	-3.77249227
C	-1.37023903	6.23408378	-4.22758993
H	-2.10261670	6.88977031	-4.72211638
H	-0.40383084	6.76136193	-4.23449532
H	-1.26009752	5.32580088	-4.83706170
C	-1.87194306	7.46387598	-1.46547441
H	-2.15628556	7.28813541	-0.41721176
H	-0.87639399	7.93450834	-1.47455648
H	-2.59166916	8.18127972	-1.88719776
C	-0.60721817	4.68287054	-1.67459018
H	-0.50480912	3.75082793	-2.24881153
H	0.38001366	5.16893308	-1.63919125
H	-0.88039169	4.41628902	-0.64224100
C	-6.18825932	5.84722154	-6.71945056
H	-5.18935419	6.21408461	-7.00014424

H	-6.91730934	6.35140539	-7.37210919
H	-6.39176104	6.16554499	-5.68706264

1−Gd

Gd	4.68423174	-2.60601055	3.20592232
Si	6.32216237	-3.94844103	6.86385807
Si	5.48443614	1.74104130	3.68413565
Si	1.86109583	-5.79108305	2.46232562
N	2.60692698	-1.55995552	1.91441443
C	4.83304773	-1.64628798	5.77661701
H	5.62637448	-0.92908091	5.96492429
C	1.56712522	-0.93528337	2.50072561
H	1.56258708	-0.92593570	3.58977393
C	3.53405387	-1.32224452	5.33018419
H	3.16492297	-0.31707685	5.13348182
C	3.64442739	-3.58955702	5.63567131
H	3.35589700	-4.63490392	5.69375140
C	5.86050795	0.03999131	2.98509384
C	4.66563245	-5.37578600	3.27007148
H	4.56318588	-5.83765388	4.24744968
C	4.93577729	-3.06326571	5.95147311
C	0.53792342	-0.31809553	1.79804013
H	-0.25265925	0.16388217	2.37118011
C	3.59369248	-5.07386068	2.38048628
C	0.53722262	-0.32346904	0.39516901
C	4.21255191	-4.53396724	1.19917367
H	3.68707236	-4.26364432	0.28390451
C	6.88073521	-0.87897245	3.38248747
H	7.42284638	-0.86065121	4.32608251
C	6.25647668	-1.45920984	1.25471486
H	6.21948274	-1.94315143	0.28166367
C	5.48624289	-0.34818666	1.66080603
H	4.75790818	0.17140472	1.04001029
C	7.12266311	-1.79736613	2.33116807

H	7.86729556	-2.59155632	2.33256886
C	2.61158089	-1.56300964	0.56481609
H	3.46165949	-2.05483201	0.09085976
C	5.89536910	-5.00746107	2.67237980
H	6.88656643	-5.14000351	3.10443763
C	2.79430256	-2.53852035	5.23915425
H	1.74643449	-2.64828105	4.96181394
C	5.61049481	-4.51624531	1.37038595
H	6.34575941	-4.18753677	0.64090797
C	1.62322646	-0.97737177	-0.21392349
H	1.72225239	-1.03797328	-1.29669212
C	6.64511888	2.97314800	2.83657596
H	7.69679710	2.73452199	3.05555627
H	6.45191611	4.00331769	3.17491738
H	6.52036893	2.94316312	1.74369684
C	8.01789237	-3.36517081	6.26188430
H	8.16823157	-3.57772331	5.19300539
H	8.81834539	-3.87207361	6.82291729
H	8.14695171	-2.28251577	6.41157490
C	6.17110102	-3.53810700	8.70376995
H	6.24537832	-2.45331559	8.87286059
H	6.96544850	-4.02864570	9.28798266
H	5.20065709	-3.87067550	9.10179055
C	5.76748200	1.86633759	5.54849407
H	5.03113420	1.29411512	6.13032107
H	5.69497123	2.91973827	5.86115358
H	6.77221960	1.51255391	5.82664026
C	1.35706116	-6.20083968	4.23671341
H	2.09350380	-6.85819877	4.72305913
H	0.39337467	-6.73342781	4.23698298
H	1.24178951	-5.30045783	4.85711099

C	3.70517687	2.25382242	3.28036462
H	3.50126022	2.17730415	2.20133066
H	3.53672118	3.30152023	3.57386845
H	2.96453010	1.63587315	3.80872726
C	0.57434574	-4.62533247	1.70721983
H	0.47640112	-3.69660935	2.28782985
H	-0.41216152	-5.11394138	1.68270629
H	0.83336680	-4.35390849	0.67226546
C	6.19630669	-5.82267683	6.65689341
H	5.19884409	-6.19619049	6.93419447
H	6.92605792	-6.31759579	7.31626945
H	6.40846814	-6.14453684	5.62721998
C	1.85679482	-7.39512929	1.45883410
H	2.13657931	-7.20685131	0.41133547
H	0.86406868	-7.87207554	1.46633792
H	2.58267420	-8.11195335	1.87129232
N	-2.60692698	1.55995552	-1.91441443
C	-1.56712522	0.93528337	-2.50072561
H	-1.56258708	0.92593570	-3.58977393
C	-0.53792342	0.31809553	-1.79804013
H	0.25265925	-0.16388217	-2.37118011
C	-0.53722262	0.32346904	-0.39516901
C	-2.61158089	1.56300964	-0.56481609
H	-3.46165949	2.05483201	-0.09085976
C	-1.62322646	0.97737177	0.21392349
H	-1.72225239	1.03797328	1.29669212
Gd	-4.68423174	2.60601055	-3.20592232
Si	-6.32216237	3.94844103	-6.86385807
Si	-5.48443614	-1.74104130	-3.68413565
Si	-1.86109583	5.79108305	-2.46232562
C	-4.83304773	1.64628798	-5.77661701

H	-5.62637448	0.92908091	-5.96492429
C	-3.53405387	1.32224452	-5.33018419
H	-3.16492297	0.31707685	-5.13348182
C	-3.64442739	3.58955702	-5.63567131
H	-3.35589700	4.63490392	-5.69375140
C	-5.86050795	-0.03999131	-2.98509384
C	-4.66563245	5.37578600	-3.27007148
H	-4.56318588	5.83765388	-4.24744968
C	-4.93577729	3.06326571	-5.95147311
C	-3.59369248	5.07386068	-2.38048628
C	-4.21255191	4.53396724	-1.19917367
H	-3.68707236	4.26364432	-0.28390451
C	-6.88073521	0.87897245	-3.38248747
H	-7.42284638	0.86065121	-4.32608251
C	-6.25647668	1.45920984	-1.25471486
H	-6.21948274	1.94315143	-0.28166367
C	-5.48624289	0.34818666	-1.66080603
H	-4.75790818	-0.17140472	-1.04001029
C	-7.12266311	1.79736613	-2.33116807
H	-7.86729556	2.59155632	-2.33256886
C	-5.89536910	5.00746107	-2.67237980
H	-6.88656643	5.14000351	-3.10443763
C	-2.79430256	2.53852035	-5.23915425
H	-1.74643449	2.64828105	-4.96181394
C	-5.61049481	4.51624531	-1.37038595
H	-6.34575941	4.18753677	-0.64090797
C	-6.64511888	-2.97314800	-2.83657596
H	-7.69679710	-2.73452199	-3.05555627
H	-6.45191611	-4.00331769	-3.17491738
H	-6.52036893	-2.94316312	-1.74369684
C	-8.01789237	3.36517081	-6.26188430

H	-8.16823157	3.57772331	-5.19300539
H	-8.81834539	3.87207361	-6.82291729
H	-8.14695171	2.28251577	-6.41157490
C	-6.17110102	3.53810700	-8.70376995
H	-6.24537832	2.45331559	-8.87286059
H	-6.96544850	4.02864570	-9.28798266
H	-5.20065709	3.87067550	-9.10179055
C	-5.76748200	-1.86633759	-5.54849407
H	-5.03113420	-1.29411512	-6.13032107
H	-5.69497123	-2.91973827	-5.86115358
H	-6.77221960	-1.51255391	-5.82664026
C	-1.35706116	6.20083968	-4.23671341
H	-2.09350380	6.85819877	-4.72305913
H	-0.39337467	6.73342781	-4.23698298
H	-1.24178951	5.30045783	-4.85711099
C	-3.70517687	-2.25382242	-3.28036462
H	-3.50126022	-2.17730415	-2.20133066
H	-3.53672118	-3.30152023	-3.57386845
H	-2.96453010	-1.63587315	-3.80872726
C	-0.57434574	4.62533247	-1.70721983
H	-0.47640112	3.69660935	-2.28782985
H	0.41216152	5.11394138	-1.68270629
H	-0.83336680	4.35390849	-0.67226546
C	-6.19630669	5.82267683	-6.65689341
H	-5.19884409	6.19619049	-6.93419447
H	-6.92605792	6.31759579	-7.31626945
H	-6.40846814	6.14453684	-5.62721998
C	-1.85679482	7.39512929	-1.45883410
H	-2.13657931	7.20685131	-0.41133547
H	-0.86406868	7.87207554	-1.46633792
H	-2.58267420	8.11195335	-1.87129232

1−Cm

Cm	4.67998870	-2.60705668	3.22755830
Si	5.48801444	1.74725697	3.67346061
Si	6.29976785	-3.94544582	6.90707369
Si	1.85126548	-5.80269670	2.45722220
C	0.53602943	-0.32376693	0.39637193
N	2.59954379	-1.56125156	1.91975616
C	0.53556403	-0.31351013	1.79952855
H	-0.25404328	0.17262672	2.37064718
C	1.61997530	-0.98321432	-0.21044199
H	1.71893832	-1.04787059	-1.29304014
C	1.56229763	-0.93091477	2.50504590
H	1.55855513	-0.91863100	3.59429874
C	2.60573401	-1.57049331	0.57036544
H	3.45412525	-2.06894475	0.09959383
C	4.81715001	-1.63840971	5.82050381
C	6.29231888	-1.46691891	1.26916694
C	7.14330523	-1.79847275	2.36023793
H	7.88613506	-2.59440090	2.37788488
C	5.87526614	0.04634290	2.98263947
C	3.51732646	-1.31165498	5.37563636
C	3.62273907	-3.58006234	5.68225094
C	6.88953464	-0.87027455	3.40143796
H	7.41918965	-0.84416911	4.35206778
C	5.51719843	-0.35233938	1.65635544
C	5.76126370	1.87872506	5.53832365
H	5.02846533	1.30031876	6.11811154
H	5.67599249	2.93177484	5.84835643
H	6.76832281	1.53708985	5.82263833
C	3.58843264	-5.09735943	2.38464298
C	4.22453458	-4.56557316	1.20913321

C	7.99467599	-3.36354960	6.30232717
H	8.79655342	-3.86551403	6.86549405
H	8.12190309	-2.28000850	6.44635509
H	8.14552642	-3.58090417	5.23465103
C	2.77461732	-2.52617010	5.28610076
C	6.16741741	-5.81823501	6.69421887
H	6.37518451	-6.13665454	5.66277253
H	5.16894518	-6.18829531	6.97218158
H	6.89582164	-6.32013724	7.34947260
C	4.91564391	-3.05581121	5.99767323
C	4.64772210	-5.39792780	3.29133114
H	4.52990641	-5.85402992	4.27001011
C	5.62080248	-4.55056123	1.39889755
C	5.88707707	-5.03863096	2.70635317
H	6.87246623	-5.17171709	3.15185062
C	3.70664041	2.24563791	3.26141458
H	3.50676378	2.16651475	2.18194932
H	3.52344626	3.29082695	3.55437757
H	2.97217438	1.61821015	3.78708382
C	6.64411665	2.98512028	2.82974098
H	7.69625393	2.74769272	3.04729979
H	6.44926962	4.01310885	3.17270510
H	6.51927199	2.96016093	1.73689459
C	1.33563019	-6.19484410	4.23186949
H	2.06432763	-6.85581108	4.72465899
H	0.36710948	-6.71821852	4.23359084
H	1.22793969	-5.28929205	4.84577063
C	1.84093596	-7.41329592	1.46475743
H	2.12319150	-7.23444478	0.41644929
H	0.84631713	-7.88577248	1.47410171
H	2.56279458	-8.13036831	1.88341314

C	0.57991916	-4.63116519	1.68577267
H	0.48109596	-3.70114060	2.26413547
H	-0.40935592	-5.11273305	1.64892695
H	0.85378730	-4.36196562	0.65420038
C	6.15407801	-3.53972422	8.74799831
H	6.94622928	-4.03665809	9.32936163
H	5.18260165	-3.86782451	9.14671114
H	6.23506518	-2.45615733	8.92066711
H	5.61201451	-0.92267660	6.00968615
H	3.14845314	-0.30611165	5.17839685
H	1.72628928	-2.63267986	5.00811699
H	3.33249667	-4.62526736	5.74172739
H	6.26632110	-1.96224151	0.30125704
H	4.79593132	0.16248306	1.02292859
H	3.71147809	-4.29753885	0.28575575
H	6.36721108	-4.22757652	0.67733827
C	-0.53602943	0.32376693	-0.39637193
N	-2.59954379	1.56125156	-1.91975616
C	-0.53556403	0.31351013	-1.79952855
H	0.25404328	-0.17262672	-2.37064718
C	-1.61997530	0.98321432	0.21044199
H	-1.71893832	1.04787059	1.29304014
C	-1.56229763	0.93091477	-2.50504590
H	-1.55855513	0.91863100	-3.59429874
C	-2.60573401	1.57049331	-0.57036544
H	-3.45412525	2.06894475	-0.09959383
Cm	-4.67998870	2.60705668	-3.22755830
Si	-5.48801444	-1.74725697	-3.67346061
Si	-6.29976785	3.94544582	-6.90707369
Si	-1.85126548	5.80269670	-2.45722220
C	-4.81715001	1.63840971	-5.82050381

C	-6.29231888	1.46691891	-1.26916694
C	-7.14330523	1.79847275	-2.36023793
H	-7.88613506	2.59440090	-2.37788488
C	-5.87526614	-0.04634290	-2.98263947
C	-3.51732646	1.31165498	-5.37563636
C	-3.62273907	3.58006234	-5.68225094
C	-6.88953464	0.87027455	-3.40143796
H	-7.41918965	0.84416911	-4.35206778
C	-5.51719843	0.35233938	-1.65635544
C	-5.76126370	-1.87872506	-5.53832365
H	-5.02846533	-1.30031876	-6.11811154
H	-5.67599249	-2.93177484	-5.84835643
H	-6.76832281	-1.53708985	-5.82263833
C	-3.58843264	5.09735943	-2.38464298
C	-4.22453458	4.56557316	-1.20913321
C	-7.99467599	3.36354960	-6.30232717
H	-8.79655342	3.86551403	-6.86549405
H	-8.12190309	2.28000850	-6.44635509
H	-8.14552642	3.58090417	-5.23465103
C	-2.77461732	2.52617010	-5.28610076
C	-6.16741741	5.81823501	-6.69421887
H	-6.37518451	6.13665454	-5.66277253
H	-5.16894518	6.18829531	-6.97218158
H	-6.89582164	6.32013724	-7.34947260
C	-4.91564391	3.05581121	-5.99767323
C	-4.64772210	5.39792780	-3.29133114
H	-4.52990641	5.85402992	-4.27001011
C	-5.62080248	4.55056123	-1.39889755
C	-5.88707707	5.03863096	-2.70635317
H	-6.87246623	5.17171709	-3.15185062
C	-3.70664041	-2.24563791	-3.26141458

H	-3.50676378	-2.16651475	-2.18194932
H	-3.52344626	-3.29082695	-3.55437757
H	-2.97217438	-1.61821015	-3.78708382
C	-6.64411665	-2.98512028	-2.82974098
H	-7.69625393	-2.74769272	-3.04729979
H	-6.44926962	-4.01310885	-3.17270510
H	-6.51927199	-2.96016093	-1.73689459
C	-1.33563019	6.19484410	-4.23186949
H	-2.06432763	6.85581108	-4.72465899
H	-0.36710948	6.71821852	-4.23359084
H	-1.22793969	5.28929205	-4.84577063
C	-1.84093596	7.41329592	-1.46475743
H	-2.12319150	7.23444478	-0.41644929
H	-0.84631713	7.88577248	-1.47410171
H	-2.56279458	8.13036831	-1.88341314
C	-0.57991916	4.63116519	-1.68577267
H	-0.48109596	3.70114060	-2.26413547
H	0.40935592	5.11273305	-1.64892695
H	-0.85378730	4.36196562	-0.65420038
C	-6.15407801	3.53972422	-8.74799831
H	-6.94622928	4.03665809	-9.32936163
H	-5.18260165	3.86782451	-9.14671114
H	-6.23506518	2.45615733	-8.92066711
H	-5.61201451	0.92267660	-6.00968615
H	-3.14845314	0.30611165	-5.17839685
H	-1.72628928	2.63267986	-5.00811699
H	-3.33249667	4.62526736	-5.74172739
H	-6.26632110	1.96224151	-0.30125704
H	-4.79593132	-0.16248306	-1.02292859
H	-3.71147809	4.29753885	-0.28575575
H	-6.36721108	4.22757652	-0.67733827

Cp′$_3$Cm

Cm	0.07652070	-0.21022934	-0.81449181
C	-1.03211121	2.26298864	-0.21518458
C	-0.16873689	2.49021130	-1.31857547
C	1.15625356	1.99506489	0.52478963
C	1.16724695	2.34746231	-0.86000621
C	-0.23047724	1.93590323	0.90258125
C	2.64551915	-0.41237799	-1.81983894
C	2.66445466	-1.14234041	-0.60964935
C	1.37275792	-2.29110245	-2.11642254
C	1.86671527	-2.30977110	-0.79145029
C	1.82170658	-1.10051889	-2.77120419
C	-1.94609965	-1.81219428	0.23029184
C	-2.56166865	-0.55360954	-0.07352195
C	-1.62198964	-2.40873490	-1.02836628
C	-2.58329548	-0.37436678	-1.47675310
C	-1.99103758	-1.52639167	-2.07142788
Si	2.61821648	2.10560918	1.70091536
Si	1.57996510	-0.66399029	-4.58494975
Si	-1.81602081	-2.63748785	1.91571849
C	3.25912011	-0.27914336	-5.35821409
H	3.15366076	-0.02489552	-6.42411016
H	3.93406230	-1.14413377	-5.27799917
H	3.74586988	0.57128146	-4.85695348
H	3.18387518	0.51484052	-2.00332808
H	3.22489707	-0.88433951	0.28706427
H	1.70879947	-3.09838909	-0.05631700
H	0.74965725	-3.05779398	-2.57099432

H	-2.11787626	2.31950230	-0.23423933
H	-0.60573077	1.73108969	1.90579442
H	-2.95904133	0.14782749	0.65814592
H	-3.00307630	0.47652166	-2.01173189
H	-0.47698323	2.77446744	-2.32460066
H	2.05778764	2.51201848	-1.46339794
C	4.23809541	2.11744025	0.72770499
H	4.38831580	1.19914984	0.14215758
H	5.09254169	2.21888890	1.41465842
H	4.27221546	2.97144621	0.03398662
C	0.46113486	0.85070459	-4.79585417
H	0.33540509	1.09190339	-5.86308837
H	0.88848044	1.73757051	-4.30387129
H	-0.54371910	0.68094240	-4.37762233
C	0.79017625	-2.13397572	-5.46859263
H	-0.19747208	-2.38171743	-5.05117667
H	1.42288269	-3.03081514	-5.39269762
H	0.65043841	-1.91063025	-6.53734923
H	-1.16586926	-3.38759874	-1.15805612
H	-1.88756335	-1.71355644	-3.13972692
C	2.62585436	0.70150197	2.96993715
H	1.68756647	0.67622157	3.54442874
H	3.44734936	0.84775606	3.68846749
H	2.76070829	-0.28757222	2.50830007
C	2.47629689	3.73656507	2.64816571
H	2.46136416	4.59199400	1.95633946
H	3.32346990	3.86992645	3.33907173
H	1.54881214	3.77055486	3.23933877

C	-1.30379911	-4.44014129	1.68313156
H	-2.03662005	-4.98509893	1.06964455
H	-1.23546622	-4.94990889	2.65637658
H	-0.32193449	-4.52811621	1.19377773
C	-3.49392571	-2.55612752	2.77797927
H	-3.81212384	-1.51552420	2.94144844
H	-3.45578975	-3.05358500	3.75962286
H	-4.26872603	-3.05133638	2.17418642
C	-0.53420689	-1.76565301	3.00412202
H	0.47712605	-1.81752935	2.57053613
H	-0.48436792	-2.23264668	4.00038149

Supplementary Note 8: References

1 Dolomanov, O. V., Bourhis, L. J., Gildea, R. J., Howard, J. A. K. & Puschmann, H. *OLEX2*: a complete structure solution, refinement and analysis program. *J. Appl. Crystallogr.* **42**, 339-341 (2009).

2 Sheldrick, G. M. *SHELXT* - Integrated space-group and crystal-structure determination. *Acta Crystallogr. Sect. A. Found. Crystallogr.* **71**, 3-8 (2015).

3 Mehdoui, T., Berthet, J. C. & Ephritikhine, M. Lanthanide(III)/actinide(III) differentiation in coordination of azine molecules to tris(cyclopentadienyl) complexes of cerium and uranium. *Dalton Trans.* **4**, 579-590 (2004).

4 Long, B. N. *et al.* Cyclopentadienyl Coordination Induces Unexpected Ionic Am−N Bonding in an Americium Bipyridyl Complex. *Nat. Commun.* **13**, 1-7 (2021).

5 Formanuik, A. *et al.* Double Reduction of 4,4'−Bipyridine and Reductive Coupling of Pyridine by Two Thorium(III) Single-Electron Transfers. *Chem. Eur. J.* **23**, 2290-2293 (2017).

6 Mehdoui, T., Berthet, J. C., Thuery, P. & Ephritikhine, M. CCDC 958634: Experimental Crystal Structure Determination. (2013).

7 Roos, B. O., Taylor, P. R. & Sigbahn, P. E. M. A Complete Active Space SCF Method (CASSCF) Using a Density Matrix Formulated Super-CI Approach. *Chem. Phys.* **48**, 157-173 (1980).

8 (Amsterdam, The Netherlands).

9 Perdew, J. P., Burke, K. & Ernzerhof, M. Perdew, Burke, and Erznzerhof Reply. *Phys. Rev. Lett.* **80**, 891 (1998).

10 Van Lenthe, E., Baerends, E. J. & Snijders, J. G. Relativistic Regular Two-Component Hamiltonians. *J. Chem. Phys.* **99**, 4597-4610 (1993).

11 Roos, B. O., Lindh, R., Malmqvist, P. A., Veryazov, V. & Widmark, P.-O. Main Group Atoms and Dimers Studied with a New Relativistic ANO Basis Set. *J. phys. Chem. A* **108**, 2851-2858 (2004).

12 Roos, B. O. *et al.* New Relativistic Atomic Natural Orbital Basis Sets for Lanthanide Atoms with Applications to the Ce Diatom and LuF_3. *J. Phys. Chem. A* **112**, 11431-11435 (2008).

13 Hess, B. A. Relativistic Electronic-Structure Calculations Employing a Two-Component No-Pair Formalism with External-Field Projection Operators. *Phys. Rev. A: At., Mol., Opt. Phys.* **33**, 3742 (1986).

14 Gagliardi, L. *et al.* Multiconfiguration Pair-Density Functional Theory: A New Way To Treat Strongly Correlated Systems. *Acc. Chem. Res.* **50**, 66-73 (2017).

15 Malmqvist, P. A., Roos, B. O. & Schimmelpfennig, B. The Restricted Active Space (RAS) State Interaction Approach with Spin-Orbit Coupling. *Chem. Phys. Lett.* **357**, 230-240 (2002).

16 Fdez. Galván, I. *et al.* OpenMolcas: From Source Code to Insight. *J. Chem. Theory. Comput.* **15**, 5925-5964 (2019).

17 Glendening, E. D., Landis, C. R. & Weinhold, F. Natural Bond Orbital Methods. *Wiley Interdiscip. Rev. Comput. Mol. Sci.* **2**, 1-42 (2012).

18 Velde, G. t. *et al.* Chemistry with ADF. *J. Comput. Chem.* **22**, 931-967 (2001).

19 Lu, T. & Chen, F. Multiwfn: A Multifunctional Wavefuntion Analyzer. *J. Comput. Chem.* **33**, 580-592 (2011).
