# Supplementary material for: Altering the spectroscopy, electronic structure, and bonding of organometallic curium(III) upon coordination of 4,4′−bipyridine
Source: Nat Commun. 2023 Jun 24;14:3774. doi: 10.1038/s41467-023-39481-7 (PMC10290646; doi:10.1038/s41467-023-39481-7)
Supplement: Supplementary file 3 — Description of Additional Supplementary Files [file 41467_2023_39481_MOESM3_ESM.pdf]

Description of Additional Supplementary Files

File Name: Supplementary Data 1
Description: Cif file for 1-Sm

File Name: Supplementary Data 2
Description: Cif file for 1-Gd

File Name: Supplementary Data 3
Description: Cif file for 1-Cm

File Name: Supplementary Data 4
Description: xyz coordinates for 1-Sm

File Name: Supplementary Data 5
Description: xyz coordinates for 1-Gd

File Name: Supplementary Data 6
Description: xyz coordinates for 1-Cm

File Name: Supplementary Data 7
Description: xyz coordinates for $CmCp'_3$
